# Single-Nuclei RNA Sequencing of 5 Regions of the Human Prenatal Brain Implicates Developing Neuron Populations in Genetic Risk for Schizophrenia

**DOI:** 10.1016/j.biopsych.2022.06.033

**Published:** 2023-01-15

**Authors:** Darren Cameron, Da Mi, Ngoc-Nga Vinh, Caleb Webber, Meng Li, Oscar Marín, Michael C. O’Donovan, Nicholas J. Bray

**Affiliations:** a MRC Centre for Neuropsychiatric Genetics & Genomics, Division of Psychological Medicine & Clinical Neurosciences, Cardiff, United Kingdom; b UK Dementia Research Institute, Cardiff University, Cardiff, United Kingdom; c Neuroscience and Mental Health Research Institute, Cardiff University, Cardiff, United Kingdom; d MRC Centre for Neurodevelopmental Disorders, King’s College London, London, United Kingdom; e Centre for Developmental Neurobiology, King’s College London, London, United Kingdom; f Tsinghua-Peking Center for Life Sciences, IDG/McGovern Institute for Brain Research, School of Life Sciences, Tsinghua University, Beijing, China

**Keywords:** Gene expression, Genetic, Genome-wide association study (GWAS), Neurodevelopment, Schizophrenia, Single-nuclei RNA sequencing

## Abstract

**Background:**

While a variety of evidence supports a prenatal component in schizophrenia, there are few data regarding the cell populations involved. We sought to identify cells of the human prenatal brain mediating genetic risk for schizophrenia by integrating cell-specific gene expression measures generated through single-nuclei RNA sequencing with recent large-scale genome-wide association study (GWAS) and exome sequencing data for the condition.

**Methods:**

Single-nuclei RNA sequencing was performed on 5 brain regions (frontal cortex, ganglionic eminence, hippocampus, thalamus, and cerebellum) from 3 fetuses from the second trimester of gestation. Enrichment of schizophrenia common variant genetic liability and rare damaging coding variation was assessed in relation to gene expression specificity within each identified cell population.

**Results:**

Common risk variants were prominently enriched within genes with high expression specificity for developing neuron populations within the frontal cortex, ganglionic eminence, and hippocampus. Enrichments were largely independent of genes expressed in neuronal populations of the adult brain that have been implicated in schizophrenia through the same methods. Genes containing an excess of rare damaging variants in schizophrenia had higher expression specificity for developing glutamatergic neurons of the frontal cortex and hippocampus that were also enriched for common variant liability.

**Conclusions:**

We found evidence for a distinct contribution of prenatal neuronal development to genetic risk for schizophrenia, involving specific populations of developing neurons within the second-trimester fetal brain. Our study significantly advances the understanding of the neurodevelopmental origins of schizophrenia and provides a resource with which to investigate the prenatal antecedents of other psychiatric and neurologic disorders.

SEE COMMENTARY ON PAGE 105

Schizophrenia is a severe psychiatric disorder characterized by profound disturbances of thought, perception, and behavior. Although the neurobiological mechanisms underlying the condition are poorly understood, it is known to have a substantial genetic component (1), of which common (>1% population frequency) risk alleles account for a sizable fraction (2,3). One of the leading theories of schizophrenia pathogenesis holds that the condition, which is typically diagnosed in early adulthood, has origins in prenatal brain development (4,5). This hypothesis, founded partly on epidemiological evidence of environmental risk factors operating in the pre- or perinatal period (6,7), has been supported in recent years by studies showing enrichment of schizophrenia risk variation within genes expressed in the prenatal human brain (8, 9, 10) and within common genetic variants associated with effects on gene regulation in fetal brain tissue (11, 12, 13, 14, 15). However, with few exceptions (16,17), these studies have been performed using bulk samples of fetal brain tissue that encompass multiple neural cell types at different stages of development.

Advances in single-cell sequencing technologies have made it possible to profile the transcriptomes of thousands of individual cells/nuclei in parallel, allowing delineation and identification of cell populations of the brain based on global gene expression (16,18, 19, 20, 21, 22). Thus defined, individual cell populations can be assessed for relevance to a given trait by testing for enrichment of trait-associated variation in the genes they express or in putative regulatory DNA elements mapped within them. This approach has provided evidence of microglial involvement in Alzheimer’s disease (22), implicated oligodendrocytes as well as monaminergic and cholinergic neurons in Parkinson’s disease (23,24), and linked common variant genetic risk of schizophrenia with gene expression in neuronal, rather than glial, cell populations in the adult brain (3,23,25).

To date, single-cell sequencing studies of the human fetal brain have largely focused on individual brain regions (16, 17, 18, 19, 20, 21), with the majority of data derived from the developing cerebral cortex (16, 17, 18, 19, 20,26). The only published studies, to our knowledge, that have used single-cell/single-nuclei sequencing data to explore cellular mediators of genetic risk of schizophrenia in the human prenatal brain have likewise focused on cortical cell populations (16,17). To provide a more extensive dataset, using uniform methodology, with which to investigate the developmental neurobiology of schizophrenia, here we report single-nuclei RNA sequencing (snRNA-seq) in 5 regions of the human second-trimester fetal brain of potential relevance to the disorder (frontal cortex [FC], ganglionic eminence [GE], hippocampus, thalamus, and cerebellum). We combine these data with results from the largest available genome-wide association study (GWAS) and exome sequencing studies of schizophrenia (3,27) to implicate specific cell populations of the human prenatal brain in genetic risk for the condition.

## Methods and Materials

### Samples

snRNA-seq was performed on brain tissue from 3 karyotypically normal human fetuses from the second trimester of gestation (2 of 14 and 1 of 15 postconception weeks, all female). Tissue was acquired from the MRC-Wellcome Trust Human Developmental Biology Resource as fresh whole brain in Hibernate-E media (Thermo Fisher Scientific). All samples were obtained through elective terminations of pregnancy, with consent from the female donors. Ethical approval for the collection and distribution of fetal material for scientific research was granted to the Human Developmental Biology Resource by the Royal Free Hospital Research Ethics Committee and North East - Newcastle and North Tyneside Research Ethics Committee. The FC, whole GE, hippocampus, thalamus, and cerebellum were dissected from both hemispheres of each sample under a light microscope and individually dounce homogenized on ice to produce cell suspensions. Aliquots of 1–10 million cells from each dissected region of each fetus were stored at −80 °C until required.

### Single-Nuclei RNA Sequencing

Cryopreserved cell suspensions were gently lysed and processed to obtain intact nuclei. snRNA-seq libraries were prepared from approximately 10,000 nuclei from each sample using the Chromium Single Cell 3ʹ Reagent kit (version 3; 10X Genomics). Quality control of libraries was performed using the 5200 Fragment Analyzer (Agilent Technologies) before sequencing on a NovaSeq 6000 (Illumina, Inc.) to a depth of at least 865 million (median = 1.01 billion) read pairs per library.

Raw sequencing data were converted into FASTQ files, aligned to the hg38 build of the human reference genome, and quantified using cellranger count (10X Genomics). A stringent quality control procedure was followed to ensure that only high-quality single-nuclei data were processed. Cells were excluded if they expressed fewer than 1000 or >5000 genes, if >5% or >10% of their transcriptome mapped to the mitochondrial genome or ribosomal genes, respectively, or if they were identified as a doublet. Genes from the mitochondrial genome or those which were expressed in fewer than 3 cells were excluded. Normalization, dimensionality reduction, clustering, and cluster visualization were carried out using Seurat version 4.0.3 (28). A supervised support vector machine classifier was used to assess cluster stability (29). We primarily assigned cell type identity to each cluster based on expression of known cell markers (Figures S1–S5 in Supplement 2) and other differentially expressed genes (Tables S2–S6 in Supplement 1). Where public single-cell RNA-seq (scRNA-seq)/snRNA-seq datasets were available from the human fetal brain region assayed (16,19,21), a cell label transfer approach was also implemented in which a reference dataset was created from the preexisting data, and the cell type labels in the reference data were projected onto the cells in the query dataset based on the similarity of their gene expression profiles (28,30). A full description of snRNA-seq analyses and assignment of cell-type identity is provided in Supplemental Methods in Supplement 2.

### MAGMA Cell Typing for Testing Enrichment of Common Variant Genetic Association

The MAGMA Celltyping package (https://github.com/neurogenomics/MAGMA_Celltyping) (25) was used to test for enrichment of schizophrenia common variant genetic associations (3) (available through: https://doi.org/10.6084/m9.figshare.14672178) within each fetal brain cell population. This package is a wrapper for the gene-set enrichment analysis MAGMA (31), and, following Bryois *et al.* (23) and Trubetskoy *et al.* (3), was used to test for enrichment of schizophrenia associations in genes with the top 10% highest expression specificity values for each fetal brain cell population. The function *map.snps.to.genes* was run to map single nucleotide polymorphisms (SNPs) in the schizophrenia GWAS (3) to genes and then to compute gene-wide association *p* values. The 1000 Genomes data (phase 3) (32) were used as reference to account for linkage disequilibrium (LD) between SNPs. The boundaries of each gene’s transcribed region were extended at a default value of of 10 kb upstream and 1.5 kb downstream. Next, for each brain region, a specificity score was calculated for each gene in each cell type by dividing a gene’s normalized (count per million) unique molecular identifier counts in one cell type by the sum of that gene’s expression in all cell types. Uninformative (i.e., sporadically expressed) genes were removed prior to this process using the function *drop.uninformative.genes*. Genes in the major histocompatibility complex region on chromosome 6 were also removed owing to its complex LD structure. These scores were then scaled and ranked into deciles so that each cell type was comparable. A linear regression was then run to test for a one-sided association between the 10% most specific genes in each cell type and the gene-level genetic association with schizophrenia. Covariates for gene size, gene density, the inverse of the minor allele count, per-gene sample size, and the log of these measures were accounted for. A total of 91 tests were performed (one for each cell population), and following Trubetskoy *et al.* (3), we primarily report enrichment (one-tailed) *p* values that exceed the Bonferroni threshold (*p* < 5.5 × 10^−4^) for these analyses. We also report enrichments with a false discovery rate (FDR) < .05. For comparison, MAGMA was also performed on GWAS data for autism (33) (available through: https://figshare.com/articles/dataset/asd2019/14671989) and human height (34) (available through: https://portals.broadinstitute.org/collaboration/giant/index.php/GIANT_consortium_data_files).

### Stratified Linkage Disequilibrium Score Regression

Following others (3,23,25), we also performed stratified linkage disequilibrium score regression (SLDSR) (35) to test the enrichment of schizophrenia SNP heritability in genes in the top expression specificity decile of each cell population. We used HapMap Project phase 3 SNPs with a minor allele frequency >5% and extended the genomic coordinates for each gene by 100 kb upstream and downstream of the transcribed region, as recommended by the SLDSR authors (35). Again, uninformative genes and genes overlapping the major histocompatibility complex region were excluded from the analysis. For each gene set, LD scores were then estimated for each SNP in relation to nearby SNPs within a 1-centimorgan window using the 1000 Genomes phase 3 reference panel files to estimate LD. Finally, schizophrenia SNP heritability was stratified for each gene set using a joint fit model accounting for SNP heritability attributable to 53 genomic annotations, including genic, enhancer, and conserved regions (baseline model version 1.2), as performed previously (3,23,25). Significance was determined empirically by calculating a *z* score based on whether schizophrenia SNP heritability was greater in each gene set compared with the baseline model annotations. As with MAGMA, we also performed SLDSR on GWAS data for autism (33) and human height (34).

### Gene Ontology Analysis

To explore biological processes mediating common variant genetic liability to schizophrenia in different populations of developing neurons, we first performed Gene Ontology (GO) enrichment analyses on genes in the top decile of expression specificity for each implicated cell type, with a background list of all genes expressed in that cell, using the DAVID Bioinformatics Resource 6.8 (36). We then used MAGMA (31) to test enrichment of schizophrenia associations (3) within genes annotated to the most significantly overrepresented GO terms for each implicated cell type to identify those processes most relevant to schizophrenia.

### Expression Analyses of Genes Containing an Excess of Rare Damaging Coding Variation

Following Singh *et al.* (27), we tested for higher expression specificity of genes carrying an excess (FDR < .05) of rare damaging coding variation in schizophrenia (27) by performing a one-sided Wilcoxon rank-sum test comparing the expression specificity score ranking of those 32 genes against the ranking of all remaining genes for each cell population. Expression specificity scores for each cell population are provided in Tables S8–S12 in Supplement 1.

## Results

### snRNA Sequencing of 5 Regions of the Human Fetal Brain

**Figure 1:**
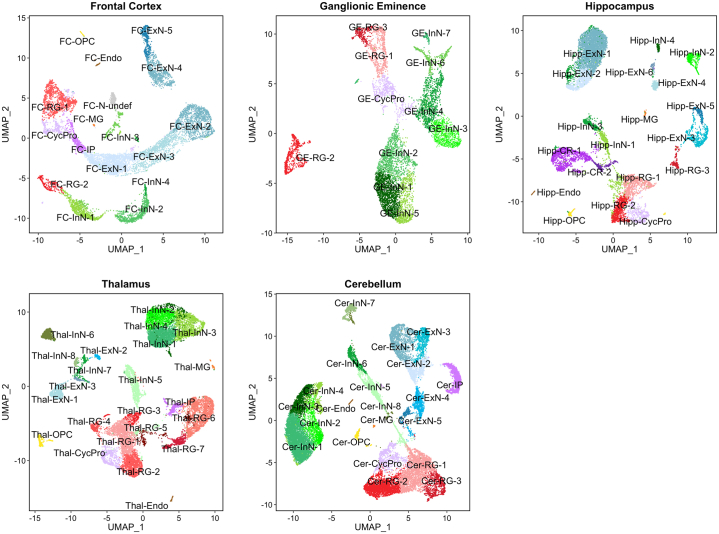
Single-nuclei RNA sequencing clusters in 5 regions of the second-trimester human fetal brain. Nuclei were clustered according to gene expression profile using shared nearest neighbor modularity optimization-based clustering in Seurat 4.0 (28) and visualized in two-dimensional space using UMAP (37). Clusters in all regions showed high stability (median F1 per region > 0.92). Clusters were annotated based on expression of known cell markers (Figures S1–S5 in Supplement 2) and other differentially expressed genes (Tables S2–S6 in Supplement 1). Cer, cerebellum; CR, Cajal-Retzius cell; CycPro, cycling progenitor cell; Endo, endothelial cell; ExN, developing excitatory neuron; FC, frontal cortex; GE, ganglionic eminence; Hipp, hippocampus; InN, developing inhibitory neuron; IP, intermediate progenitor; MG, microglia; N-undef, neuron of undefined class; OPC, oligodendrocyte precursor cell; RG, radial glia; Thal, thalamus; UMAP, Uniform Manifold Approximation and Projection.

We performed snRNA-seq on the 5 dissected brain regions from 3 human fetuses from the second trimester of gestation. After strict quality control, we retained snRNA-seq data from 13,597 nuclei from the FC, 11,310 nuclei from the GE, 12,594 nuclei from the hippocampus, 17,283 nuclei from the thalamus, and 22,652 nuclei from the cerebellum. Nuclei were clustered according to gene expression profile using shared nearest neighbor modularity optimization-based clustering (26). We thus identified 17 transcriptionally distinct clusters of nuclei in the fetal FC, 11 in the GE, 19 in the hippocampus, 23 in the thalamus, and 21 in the cerebellum (Figure 1). The defined clusters in all regions showed high stability (median F1 per region > 0.92) (Table S1 in Supplement 1).

Clusters were annotated based on expression of known cell markers (Figures S1–S5 in Supplement 2) and other differentially expressed genes (Tables S2–S6 in Supplement 1). In addition to populations of radial glia (markers: *GLI3*, *TNC*), intermediate progenitors (marker: *EOMES*), oligodendrocyte precursor cells (marker: *OLIG1*), endothelial cells (marker: *ITM2A*), and microglia (marker: *C3*), we were able to define a variety of developing neuron populations appropriate for each region. Thus, in the FC, we found developing excitatory (glutamatergic) neuron populations differentially expressing markers of upper cortical layers, such as *TLE3* and *LHX2* (FC-ExN-1, FC-ExN-2, and FX-ExN-3), or markers of deep cortical layers, such as *CRYM* and *FEZF2* (FC-ExN-4 and FC-ExN-5), as well as developing GABAergic (gamma-aminobutyric acidergic) interneuron populations expressing *CALB2* (FC-InN-1) or *SST* (FC-InN-4). In the GE, a transitory structure of the prenatal brain that gives rise to cortical and subcortical inhibitory neurons (38), we observed interneuron populations expressing the medial GE marker *LHX6* (GE-InN-1, GE-InN-2, and GE-InN-5) or the caudal GE marker *PROX1* (GE-InN-3), in addition to a population (GE-InN-6) that we predict to be developing medium spiny neurons from the lateral GE based on expression of *SIX3* and *TSHZ1* (38). In the hippocampus, we identified 2 populations highly expressing the Cajal-Retzius cell marker *RELN* (Hipp-CR-1 and Hipp-CR-2), 2 excitatory neuron populations expressing the dentate gyrus granule cell marker *PROX1* (Hipp-ExN-3 and Hipp-ExN-6), 4 excitatory neuron populations expressing the CA3 marker *GRIK4* (Hipp-ExN-1, Hipp-ExN-2, Hipp-ExN-4, and Hipp-ExN-5), and populations of interneuron predicted to derive from the medial (Hipp-InN-2) or caudal GE (Hipp-InN-1 and Hipp-InN-4). In the thalamus, we discerned 3 developing glutamatergic neuron populations expressing *SLC17A6* (Thal-ExN-1, Thal-ExN-2, and Thal-ExN-3), along with 8 GABAergic populations expressing *SLC6A1* and/or *GAD2* (Thal-InN-1, Thal-InN-2, Thal-InN-3, Thal-InN-4, Thal-InN-5, Thal-InN-6, Thal-InN-7, and Thal-InN-8). In the cerebellum, we observed 5 populations expressing markers of (excitatory) granule neurons such as *RBFOX3* and *RELN* (Cer-ExN-1, Cer-ExN-2, Cer-ExN-3, Cer-ExN-4, and Cer-ExN-5) as well as 4 populations of (inhibitory) Purkinje cells marked by expression of *CA8* and *ITPR1* (Cer-InN-1, Cer-InN-2, Cer-InN-3, Cer-InN-4). There was strong concordance between our annotations and predictions using data from previous scRNA-seq/snRNA-seq studies of the human fetal brain (16,19,21) Figures S6–S8 in Supplement 2).

### Common Risk Alleles for Schizophrenia Are Enriched in Genes With High Expression Specificity for Developing Neurons of the Fetal Brain

Single-cell/single-nuclei data from mice and humans have indicated enrichment of common schizophrenia risk variation in genes with high expression specificity for populations of mature neurons in the adult brain (3,23,25). To investigate cells of the prenatal human brain mediating common variant genetic liability for schizophrenia, we similarly determined the top decile of gene expression specificity for each of the identified fetal brain cell populations (Table S7 in Supplement 1) and used 2 different statistical methods [MAGMA (31) and SLDSR (35)] to assess the enrichment of schizophrenia genetic risk (3) within those gene loci.

**Figure 2:**
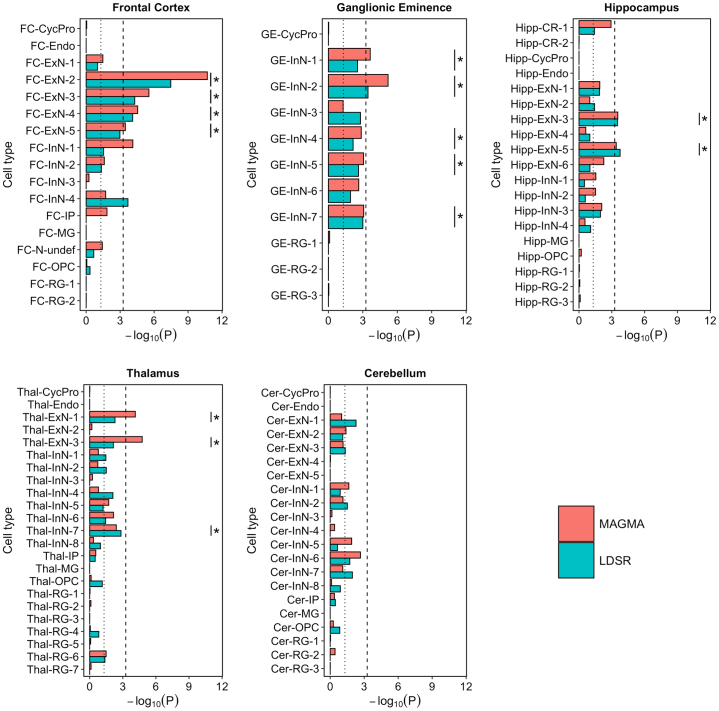
Log_10_*p* values for enrichment of schizophrenia genetic associations (3) in genes in the top expression specificity decile for each identified fetal brain cell population using MAGMA and stratified LDSR. The dotted vertical line indicates nominal (*p* < .05) significance and the dashed vertical line indicates the Bonferroni-corrected *p* value threshold for 91 tested cell populations (*p* < 5.5 × 10^−4^). ∗Cell populations in which enrichments in both MAGMA and stratified LDSR are false discovery rate < 0.05. Cer, cerebellum; CR, Cajal-Retzius cell; CycPro, cycling progenitor cell; Endo, endothelial cell; ExN, developing excitatory neuron; FC, frontal cortex; GE, ganglionic eminence; Hipp, hippocampus; InN, developing inhibitory neuron; IP, intermediate progenitor; LDSR, linkage disequilibrium score regression; MG, microglia; N-undef, neuron of undefined class; OPC, oligodendrocyte precursor cell; RG, radial glia; Thal, thalamus.

Both MAGMA and SLDSR showed significant enrichment (exceeding the Bonferroni threshold of *p* < 5.5 × 10^−4^) of schizophrenia genetic risk in genes with high expression specificity for 6 fetal cell populations, all of which were annotated as developing neurons (Figure 2). The most significant enrichment was observed for genes with high expression specificity for FC-ExN-2 (*P*_MAGMA_ = 1.98 × 10^−11^; *P*_sLDSR_ = 3.33 × 10^−8^), a maturing upper layer pyramidal neuron of the FC. Schizophrenia genetic risk was also prominently enriched in genes with high expression specificity for another upper layer glutamatergic neuron of the FC (FC-ExN-3), a deep layer excitatory neuron of the FC (FC-ExN-4), a developing interneuron population within the (medial) GE (GE-InN-2) and 2 developing glutamatergic neuron populations from the hippocampus (Hipp-ExN-3, expressing markers of dentate gyrus granule cells, and Hipp-ExN-5, expressing markers of CA3 pyramidal cells). A further 8 fetal cell populations were enriched for schizophrenia genetic liability at the less conservative FDR < .05 threshold, including another deep layer excitatory neuron of the FC (FC-ExN-5), 4 additional developing inhibitory neuron populations of the GE (GE-InN-1, GE-InN-4, GE-InN-5, and GE-InN-7), and 3 neuronal populations of the thalamus (Thal-ExN-1, Thal-ExN-3, and Thal-InN-7). We found little evidence of enrichment of schizophrenia common variant liability in genes with high expression specificity for progenitor or glial cells in any region. This pattern of neuronal, rather than progenitor, enrichment was maintained even when large differences in gene boundary were applied (Figures S9 and S10 in Supplement 2).

For comparison, we tested enrichment of the common genetic variation associated with autism (33) and human height (34) as neurodevelopmental and nonbrain phenotypes, respectively, within the same high expression specificity genes for each fetal cell population. Although autism GWASs currently provide limited statistical power for these analyses, we observed nominally significant (*p* < .05) enrichment of associations in the deep layer excitatory neuron FC-ExN-4 of the frontal cortex and oligodendrocyte precursor Thal-OPC of the thalamus by both MAGMA and SLDSR (Figure S11 in Supplement 2). In contrast, despite strong statistical power, variants influencing human height were not enriched in any neural population; instead, MAGMA indicated significant enrichment within genes in the top expression specificity decile for endothelial cells (Figure S12 in Supplement 2).

### Independence of Schizophrenia Genetic Association Between Implicated Cell Types of the Fetal Brain

**Figure 3:**
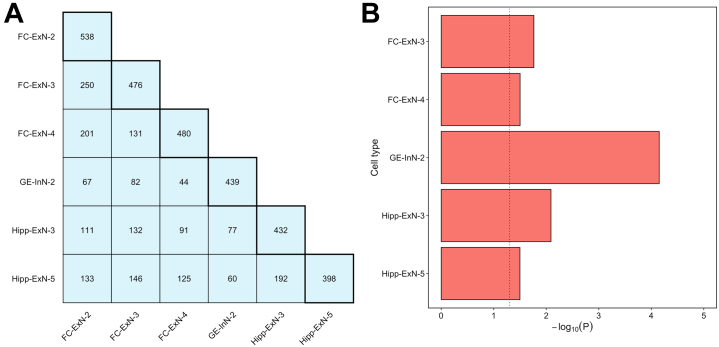
Independence of schizophrenia genetic association between implicated cell types of the fetal brain. **(A)** Number of genes exhibiting nominally significant genetic association with schizophrenia (MAGMA genewise *p* < .05) shared between the top expression specificity deciles of each fetal cell population implicated in the disorder at a level exceeding the Bonferroni significance threshold in both MAGMA and stratified linkage disequilibrium score regression analysis. The total number of schizophrenia-associated genes (MAGMA genewise *p* < .05) in the top expression specificity decile of each cell type is shown in boxes with bold borders. **(B)** MAGMA −log_10_*p* values for enrichment of schizophrenia genetic associations (3) in genes in the top expression specificity deciles of 5 fetal brain cell populations implicated in the disorder, conditioning on genes in the top expression specificity decile of the most significantly enriched cell type (FC-ExN-2). The dotted vertical line indicates *p* = .05. ExN, developing excitatory (glutamatergic) neuron; FC, frontal cortex; GE, ganglionic eminence; Hipp, hippocampus; InN, developing inhibitory (GABAergic) neuron.

We next investigated the extent to which observed enrichments of schizophrenia risk alleles in different populations of developing neurons represent independent signals. Figure 3A shows the overlap of nominally significant schizophrenia-associated genes (MAGMA genewise *p* < .05) in the top expression specificity decile of the 6 cell populations of the fetal brain that we implicate in the disorder at the Bonferroni significance threshold. Cell populations generally shared less than half of the schizophrenia-associated genes in their top specificity deciles with any other implicated cell population, suggesting partly independent enrichment signals. We formally tested for enrichment of schizophrenia genetic association independent of genes shared with the most strongly enriched cell type FC-ExN-2 by repeating the MAGMA cell-specific expression analyses of the 5 other implicated cell types, conditioning on genes in the top expression specificity decile of FC-ExN-2 (Figure 3B). All implicated fetal cell populations remained significantly (*p* < .05) enriched for genetic association with schizophrenia when genes shared with FC-ExN-2 were accounted for.

### Enrichment of Schizophrenia Genetic Risk Within Developing Neurons of the Fetal Brain Is Largely Independent of Genetic Enrichments in Adult Neurons

**Figure 4:**
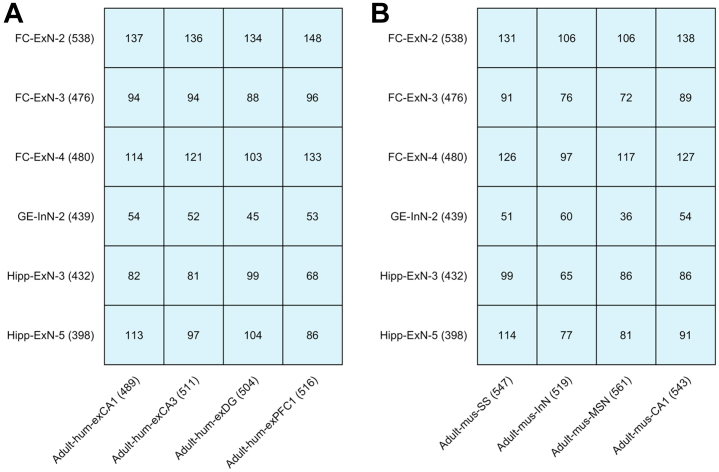
Overlap of schizophrenia-associated genes in the top expression specificity decile of implicated neuron populations of the fetal and adult human brain. **(A)** Number of genes exhibiting nominally significant genetic association with schizophrenia (MAGMA genewise *p* < .05) shared between the top expression specificity deciles of each fetal cell population (implicated in the disorder at a level exceeding the Bonferroni significance threshold in both the MAGMA and stratified linkage disequilibrium score regression analysis) and each similarly implicated adult human cell population (3). **(B)** Number of genes exhibiting nominally significant genetic association with schizophrenia (MAGMA genewise *p* < .05) shared between the top expression specificity deciles of each fetal cell population (implicated in the disorder at a level exceeding the Bonferroni significance threshold in both the MAGMA and stratified linkage disequilibrium score regression analysis) and each similarly implicated adult mouse cell population. Adult-hum-exCA1, adult human hippocampal CA1 pyramidal neurons; Adult-hum-exCA3, adult human hippocampal CA3 pyramidal neurons; Adult-hum-exDG, adult human dentate gyrus granule neurons; Adult-hum-exPFC1, adult human frontal cortex pyramidal neurons; Adult-mus-CA1, adult mouse hippocampal CA1 pyramidal neurons; Adult-mus-InN, adult mouse cortical interneurons; Adult-mus-MSN, adult mouse striatal medium spiny neurons; Adult-mus-SS, adult mouse cortical somatosensory pyramidal neurons; ExN, developing excitatory (glutamatergic) neuron; FC, frontal cortex; GE, ganglionic eminence; Hipp, hippocampus; InN, developing inhibitory (GABAergic) neuron.

**Figure 5:**
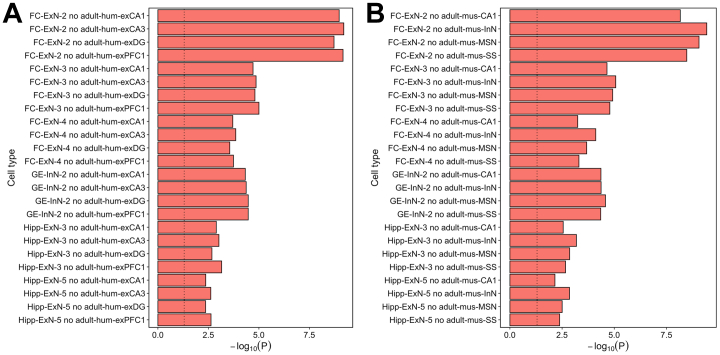
MAGMA −log_10_*p* values for enrichment of common variant risk for schizophrenia (3) in implicated fetal neuron populations, conditioning on genes in the top expression specificity decile of each implicated adult neuron population. The dotted vertical line indicates *p* = .05. **(A)** MAGMA −log_10_*p* values for enrichment of schizophrenia common variant liability in fetal neuron populations (implicated in the disorder at a level exceeding the Bonferroni significance threshold in both the MAGMA and stratified linkage disequilibrium score regression analysis), conditioning on genes in the top expression specificity decile of each similarly implicated adult human cell population (3). **(B)** MAGMA −log_10_*p*-values for enrichment of schizophrenia common variant liability in fetal neuron populations (implicated in the disorder at a level exceeding the Bonferroni significance threshold in both the MAGMA and stratified linkage disequilibrium score regression analysis), conditioning on genes in the top expression specificity decile of each similarly implicated adult mouse cell population (3). Adult-hum-exCA1, adult human hippocampal CA1 pyramidal neurons; Adult-hum-exCA3, adult human hippocampal CA3 pyramidal neurons; Adult-hum-exDG, adult human dentate gyrus granule neurons; Adult-hum-exPFC1, adult human frontal cortex pyramidal neurons; Adult-mus-CA1, adult mouse hippocampal CA1 pyramidal neurons; Adult-mus-InN, adult mouse cortical interneurons; Adult-mus-MSN, adult mouse striatal medium spiny neurons; Adult-mus-SS, adult mouse cortical somatosensory pyramidal neurons; ExN, developing excitatory (glutamatergic) neuron; FC, frontal cortex; GE, ganglionic eminence; Hipp, hippocampus; InN, developing inhibitory (GABAergic) neuron.

We then assessed the extent to which enrichment of schizophrenia genetic risk in developing neuronal populations of the fetal brain can be explained by genes shared with neurons of the adult brain that have been implicated in the disorder through the same methods (3,23,25). Figure 4 shows the overlap of nominally significant schizophrenia-associated genes (MAGMA genewise *p* < .05) in the top expression specificity decile of the 6 fetal neuron populations that we implicate in the disorder at the Bonferroni threshold with those in the top expression specificity deciles of neuronal populations implicated in schizophrenia using snRNA-seq data from the adult human brain (FC pyramidal neurons, dentate gyrus granule neurons, hippocampal CA1 pyramidal neurons, and hippocampal CA3 pyramidal neurons) and scRNA-seq data from adolescent mouse brain (hippocampal CA1 pyramidal neurons, striatal medium spiny neurons, cortical interneurons, and cortical somatosensory pyramidal neurons). None of the fetal cell populations shared more than 29% of schizophrenia-associated genes with any adult neuron, suggesting largely independent enrichments. We confirmed this by repeating the MAGMA cell-specific expression analyses, conditioning on genes in the top expression specificity decile of each implicated adult neuron population (Figure 5). All implicated fetal cell populations remained highly enriched (maximum *p* < .007) for schizophrenia genetic association, indicating that variants acting through genes expressed in the developing neurons of the prenatal brain make a distinct contribution to the risk of schizophrenia.

### Biological Processes Mediating Common Variant Genetic Association With Schizophrenia in Cells of the Developing Brain

**Figure 6:**
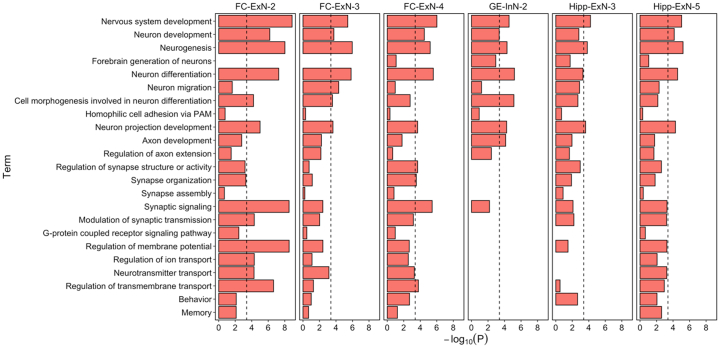
MAGMA −log_10_*p* values for enrichment of schizophrenia genetic associations (3) within overrepresented gene ontology terms belonging to the top expression specificity decile of each implicated cell population. The dashed line indicates the Bonferroni-corrected *p*-value threshold for 121 tests (*p* < 4.1 × 10^−4^). ExN, developing excitatory (glutamatergic) neuron; FC, frontal cortex; GE, ganglionic eminence; Hipp, hippocampus; InN, developing inhibitory (GABAergic) neuron.

To better understand the biological processes mediating genetic association with schizophrenia in cells of the second-trimester fetal brain, we first performed GO enrichment analyses on genes in the top decile of expression specificity of the 6 cell populations implicated in the disorder by MAGMA and SLDSR at the Bonferroni threshold. As expected, cell-specific gene sets were significantly enriched (FDR < .05) for terms relating to neuronal development, although we note that genes with high expression specificity for implicated excitatory neuron populations were also enriched for mature neuronal functions such as synaptic signaling and regulation of membrane potential (Figure S13 in Supplement 2). To determine which of these processes are relevant to schizophrenia genetic liability, we used MAGMA to test for enrichment of schizophrenia associations (3) within genes annotated to overrepresented GO terms in the top expression specificity decile of each cell population (Figure 6). Consistent with the important role of neuronal development in schizophrenia susceptibility, risk alleles were significantly enriched (at a level exceeding the Bonferroni *p* value threshold) within genes annotated to terms such as “neurogenesis,” “neuron differentiation,” and “neuron projection development” belonging to all 6 cell populations. For genes with high expression specificity for FC-ExN-2 and FC-ExN-4, schizophrenia genetic associations were additionally enriched for synaptic signaling, which is likely to index a later postnatal risk mechanism for the disorder. These analyses thus suggest several mechanisms by which common alleles confer risk for schizophrenia within developing neurons.

### Cellular Expression Specificity of Genes Implicated in Schizophrenia Through Rare Coding Variation

**Figure 7:**
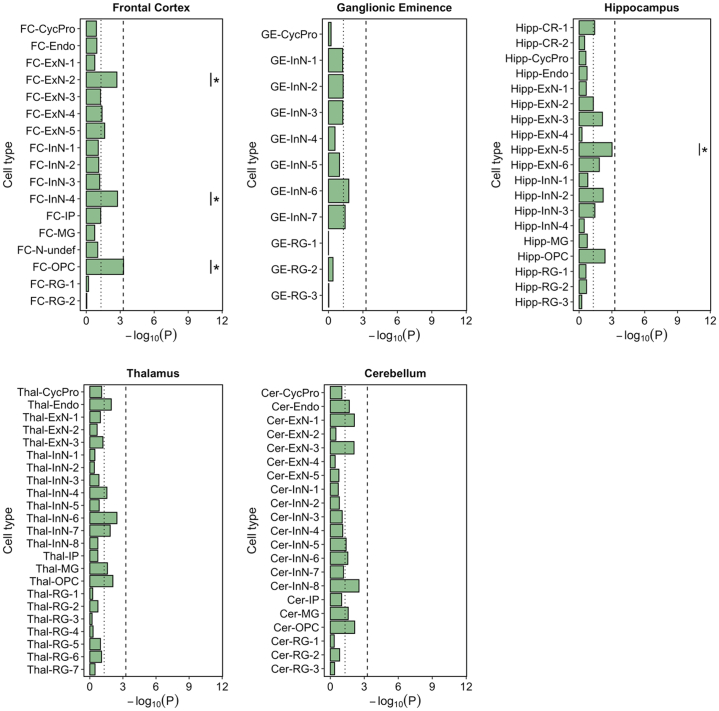
Wilcoxon rank-sum test −log_10_*p* values for higher gene expression specificity of 32 genes carrying an excess (false discovery rate < 0.05) of rare damaging coding variation in schizophrenia (27) in cell populations of the human fetal brain. The dotted vertical line indicates nominal (*p* < .05) significance, the dashed vertical line indicates the Bonferroni-corrected *p*-value threshold for 91 tested cell populations (*p* < 5.5 × 10^−4^) and ∗ indicate cell populations in which 32 genes have higher expression specificity at false discovery rate < 0.05. CR, Cajal-Retzius cell; CycPro, cycling progenitor cell; Endo, endothelial cell; ExN, developing excitatory neuron; InN, developing inhibitory neuron; IP, intermediate progenitor; MG, microglia; N-undef, neuron of undefined class; OPC, oligodendrocyte precursor cell; RG, radial glia.

A recent large-scale exome sequencing study identified 10 genes containing an exome-wide significant excess of rare damaging coding variants in schizophrenia, with 32 such genes at FDR < .05 (27). Using scRNA-seq data from the adolescent mouse nervous system (39), the authors reported that the 32 FDR < .05 genes had significantly higher expression specificity for (brain-derived) neuronal populations. Applying a similar methodology, we found higher expression specificity of these 32 genes in 4 cell populations of the human fetal brain at FDR < .05 (Figure 7), namely FC-ExN-2, FC-InN-4, FC-OPC, and Hipp-ExN-5. We note that FC-ExN-2 and Hipp-ExN-5 also display enrichment of common variant genetic liability for schizophrenia, suggesting points of biological convergence. We performed an identical analysis of 78 genes implicated in autism through exome sequencing (40) at FDR < .05, finding expression enrichment in a variety of developing cell populations (Figure S14 in Supplement 2), consistent with prior literature (16,40).

## Discussion

By combining single-nuclei RNA sequencing of 5 regions of the human fetal brain with recent large-scale genomic data for schizophrenia (3,27), we implicate specific neuronal populations within the developing FC, GE, and hippocampus in genetic liability to the disorder.

To our knowledge, only 2 other published studies have used human single-cell sequencing data to explore cellular mediators of genetic risk of schizophrenia in the prenatal brain, both of which focused on the developing cerebral cortex. The first, by Polioudakis *et al.* (16), used SLDSR to test for the enrichment of schizophrenia common variant liability in regions of open chromatin (an index of regulatory genomic regions) associated with genes enriched in specific cell types of the second-trimester fetal neocortex (gestation week 17–18). Consistent with our findings, the authors found enrichment of schizophrenia genetic risk in regulatory genomic sites associated with developing glutamatergic populations, although significant enrichments were also observed in sites associated with a variety of other cell types, including radial glia, microglia, and oligodendrocyte precursors (the latter also enriched for genes containing an excess of rare damaging coding mutations in schizophrenia in this study). More recently, Ziffra *et al.* (17) used SLDSR to test the enrichment of schizophrenia common variant genetic risk in predicted enhancers among cell-specific open chromatin regions of the midgestation human cerebral cortex. While implicating no progenitor or glial cell population, the authors found an enrichment of schizophrenia-associated variation in enhancers attributed to upper layer excitatory neurons, consistent with this study, as well as cortical interneurons predicted to derive from the caudal GE.

Although studies based on single-cell sequencing data have implicated neuronal populations of the adult hippocampus in genetic risk for schizophrenia (3,23,25), to our knowledge, our study is the first to implicate cell populations of the prenatal hippocampus in genetic liability to the disorder. Our finding that schizophrenia genetic risk is enriched in developing glutamatergic neurons of the FC and hippocampal formation supports the view that the disorder has origins in the initial formation of neuronal connectivity within those regions (41, 42, 43, 44) and may partly explain reductions in synaptic markers observed in those areas in postmortem studies of schizophrenia (44,45). Our observation that common variant genetic risk of schizophrenia is independently enriched within genes with high expression specificity for at least one interneuron population of the GE provides evidence that early GABAergic neuron development is also perturbed in the disorder (46,47). At a more relaxed significance threshold of FDR < .05, we additionally implicate neuronal populations of the developing thalamus in common variant liability to schizophrenia.

Limitations of this study are that we focused on one developmental time point and from only female samples. Future studies could also extend the range of brain regions to include others of potential relevance to schizophrenia. Although we were able to use GWAS and exome sequencing data from many thousands of participants (3,27), still larger sample sizes are likely to bring greater resolution of relevant cell types, as illustrated in gains observed through adult brain cell specific–enrichment analyses performed using successive schizophrenia GWASs of the Psychiatric Genomics Consortium (PGC) (3). We therefore provide our cellular expression data for future studies exploring the prenatal antecedents of schizophrenia and other brain-related traits.

## Supplementary Material

Supplementary Material

Key Resource Table
