# Supplementary material for: Single-Nuclei RNA Sequencing of 5 Regions of the Human Prenatal Brain Implicates Developing Neuron Populations in Genetic Risk for Schizophrenia
Source: Biol Psychiatry. 2023 Jan 15;93(2):157–66. doi: 10.1016/j.biopsych.2022.06.033 (PMC10804933; doi:10.1016/j.biopsych.2022.06.033)
Supplement: Supplementary Material [file mmc2.pdf]

Single Nuclei RNA Sequencing of 5 Regions of the Human Prenatal Brain Implicates Developing Neuron Populations in Genetic Risk for Schizophrenia

Supplementary Information

Supplementary Figures

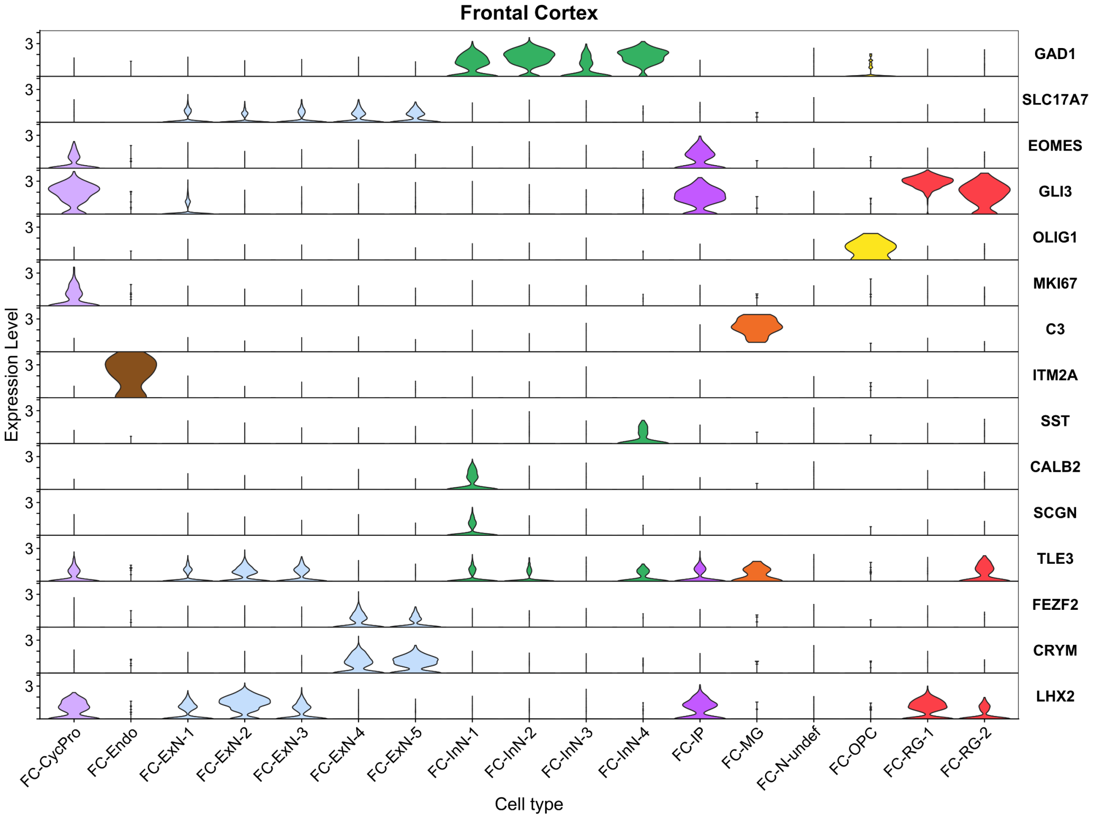

Figure S1. Violin plots of cell marker gene expression across clusters identified in the fetal frontal cortex. ExN = developing excitatory neuron; InN = developing inhibitory neuron; N-undef = neuron of undefined class; IP = intermediate progenitor; RG = radial glia; CycPro = cycling progenitor cells; OPC = oligodendrocyte precursor cell; Endo = endothelial cell; MG = microglia.

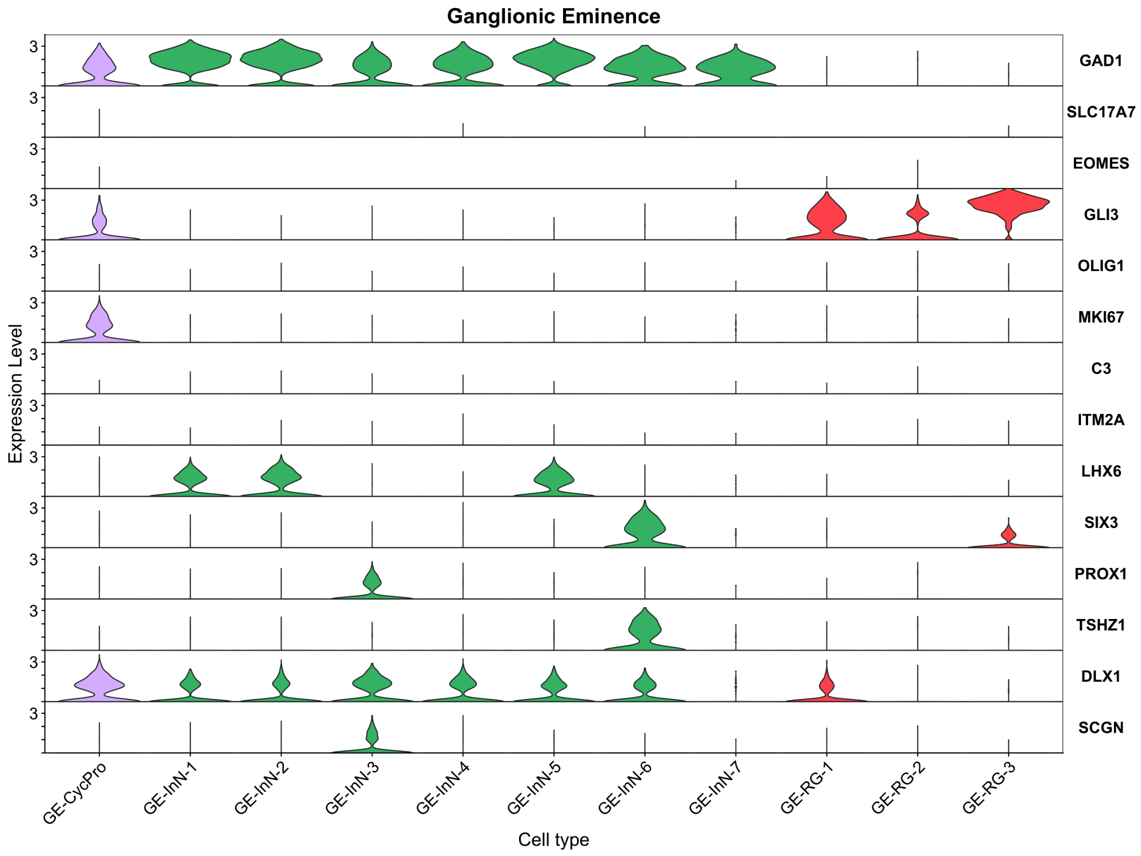

Figure S2. Violin plots of cell marker gene expression across clusters identified in the ganglionic eminence. InN = developing inhibitory neuron; IP = intermediate progenitor; RG = radial glia; CycPro = cycling progenitor cells

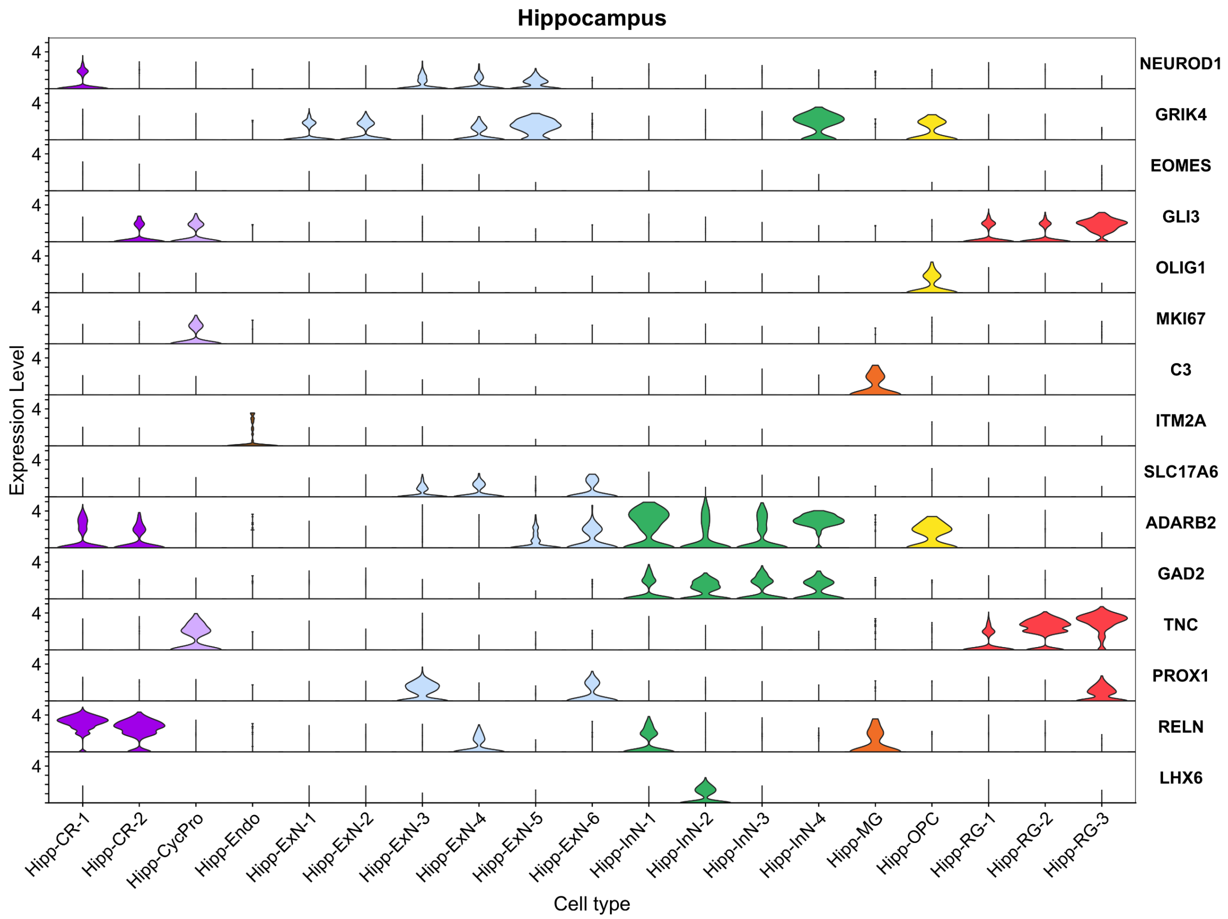

Figure S3. Violin plots of cell marker gene expression across clusters identified in the fetal hippocampus. ExN = developing excitatory neuron; InN = developing inhibitory neuron; CR = Cajal-Retzius cell; RG = radial glia; CycPro = cycling progenitor cells; OPC = oligodendrocyte precursor cell; Endo = endothelial cell; MG = microglia.

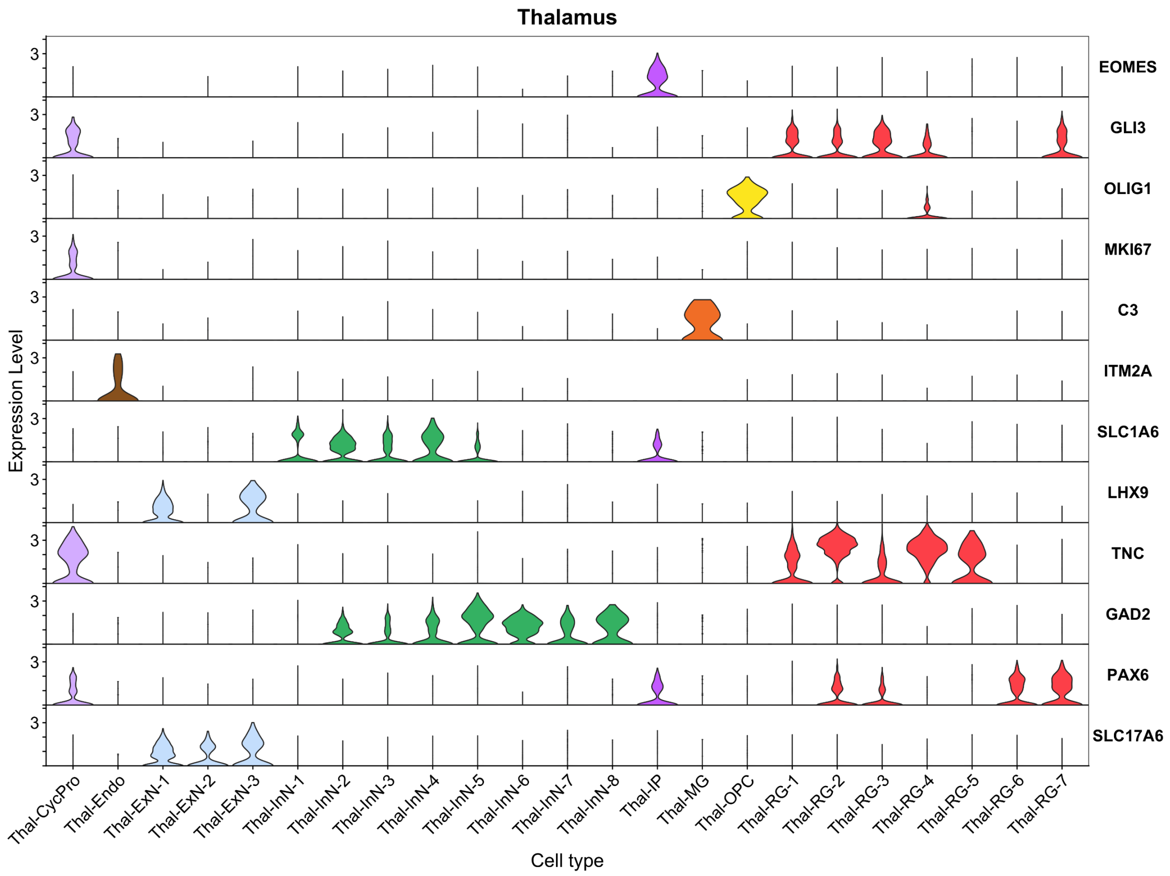

Figure S4. Violin plots of cell marker gene expression across clusters identified in the fetal thalamus. ExN = developing excitatory neuron; InN = developing inhibitory neuron; IP = intermediate progenitor; RG = radial glia; CycPro = cycling progenitor cells; OPC = oligodendrocyte precursor cell; Endo = endothelial cell; MG = microglia.

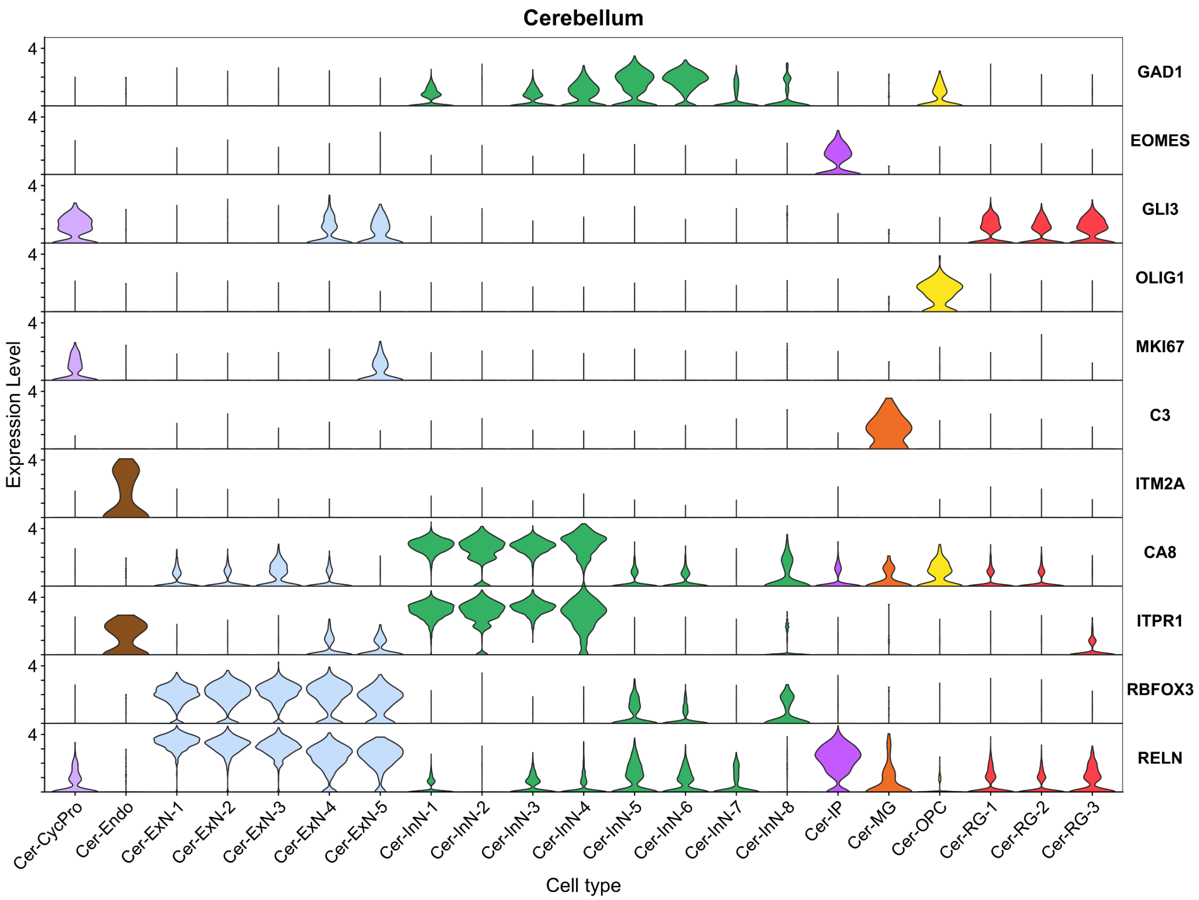

Figure S5. Violin plots of cell marker gene expression across clusters identified in the fetal cerebellum. ExN = developing excitatory neuron; InN = developing inhibitory neuron; IP = intermediate progenitor; RG = radial glia; CycPro = cycling progenitor cells; OPC = oligodendrocyte precursor cell; Endo = endothelial cell; MG = microglia.

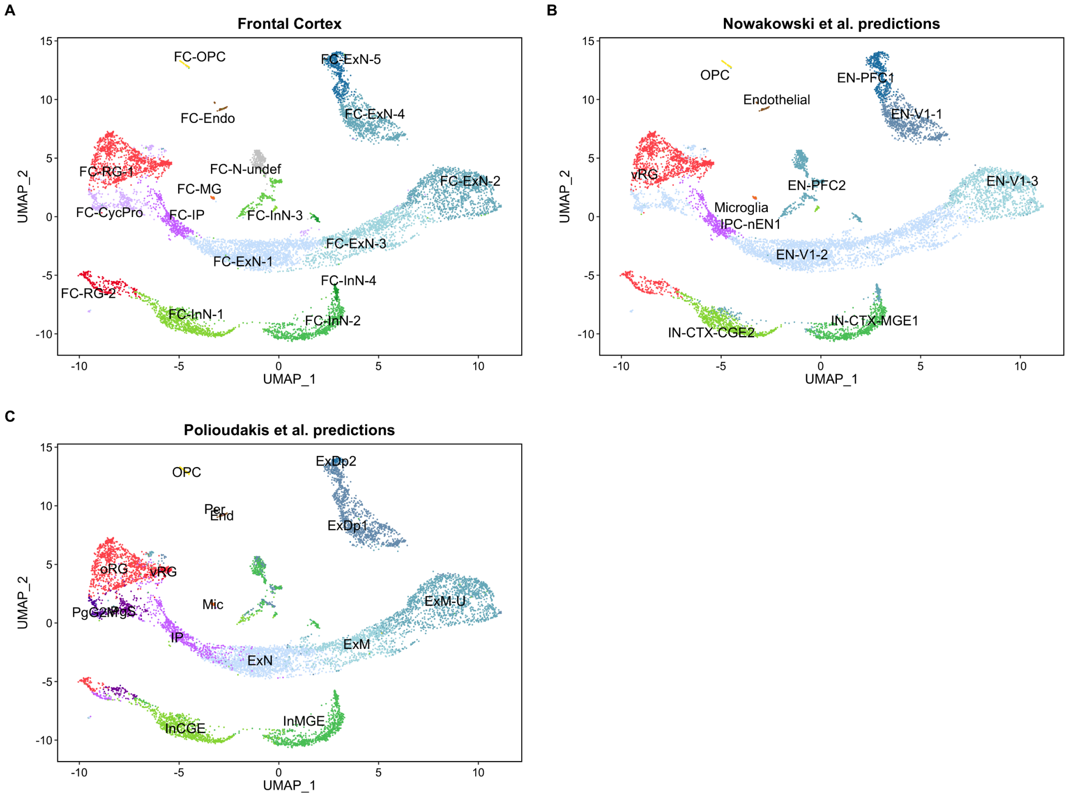

Figure S6. Cluster annotations for fetal frontal cortex. A) Labels used in this study. ExN = developing excitatory neuron; InN = developing inhibitory neuron; N-undef = neuron of undefined class; IP = intermediate progenitor; RG = radial glia; CycPro = cycling progenitor cells; OPC = oligodendrocyte precursor cell; Endo = endothelial cell; MG = microglia. FC-InN-1 expresses the caudal ganglionic eminence marker *PROX1*, whereas FC-InN-2 and FC-InN-3 express the medial ganglionic eminence marker *LHX6*. B) Predictions based on data from the study of Nowakowski et al (2017). EN = excitatory neuron; IN = inhibitory neuron; IPC = intermediate progenitor; RG = radial glia; OPC = oligodendrocyte precursor cell; V1 = primary visual cortex; PFC = prefrontal cortex; MGE = medial ganglionic eminence; CGE, caudal ganglionic eminence; C) Predictions based on data from the study of Polioudakis et al (2019). ExN = Migrating excitatory; ExM = Maturing excitatory; ExM-U = Maturing excitatory upper enriched; ExDp1 = Excitatory deep layer 1; ExDp2 = Excitatory deep layer 2; InMGE = Interneuron MGE; InCGE = Interneuron CGE; OPC = oligodendrocyte precursor cell; RG = Radial glia; End = Endothelial; Mic = microglia; PgG2M = Cycling Progenitors (G2/M phase); PgS = Cycling progenitors (S phase).

References

Nowakowski TJ, Bhaduri A, Pollen AA, Alvarado B, Mostajo-Radji MA, Di Lullo E, et al. (2017): Spatiotemporal gene expression trajectories reveal developmental hierarchies of the human cortex. *Science* 358: 1318–1323.

Polioudakis D, de la Torre-Ubieta L, Langerman J, Elkins AG, Shi X, Stein JL, et al. (2019): A Single-Cell Transcriptomic Atlas of Human Neocortical Development during Mid-gestation. *Neuron* 103: 785–801.

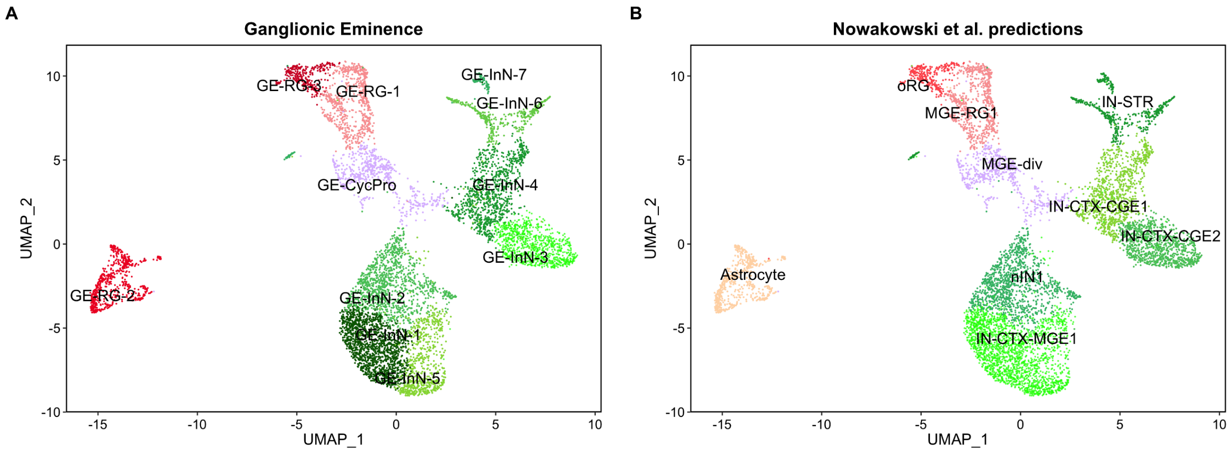

Fig. S7. Cluster annotations for ganglionic eminence. A) Labels used in this study. InN = developing inhibitory neuron; RG = radial glia; CycPro = cycling progenitor cells. GE-InN-1, GE-InN-2 and GE-InN-5 express the medial ganglionic eminence marker *LHX6*, whereas GE-InN-3 expresses the caudal ganglionic eminence marker *PROX1*. GE-InN-6 is predicted to be developing medium spiny neurons from the lateral ganglionic eminence based on expression of *SIX3* and *TSHZ1*. Predictions based on data from the study of Nowakowski et al (2017). IN = inhibitory neuron; IRG = radial glia; MGE = medial ganglionic eminence; CGE, caudal ganglionic eminence; MGE-div = medial ganglionic eminence dividing.

Reference

Nowakowski TJ, Bhaduri A, Pollen AA, Alvarado B, Mostajo-Radji MA, Di Lullo E, et al. (2017): Spatiotemporal gene expression trajectories reveal developmental hierarchies of the human cortex. *Science* 358: 1318–1323.

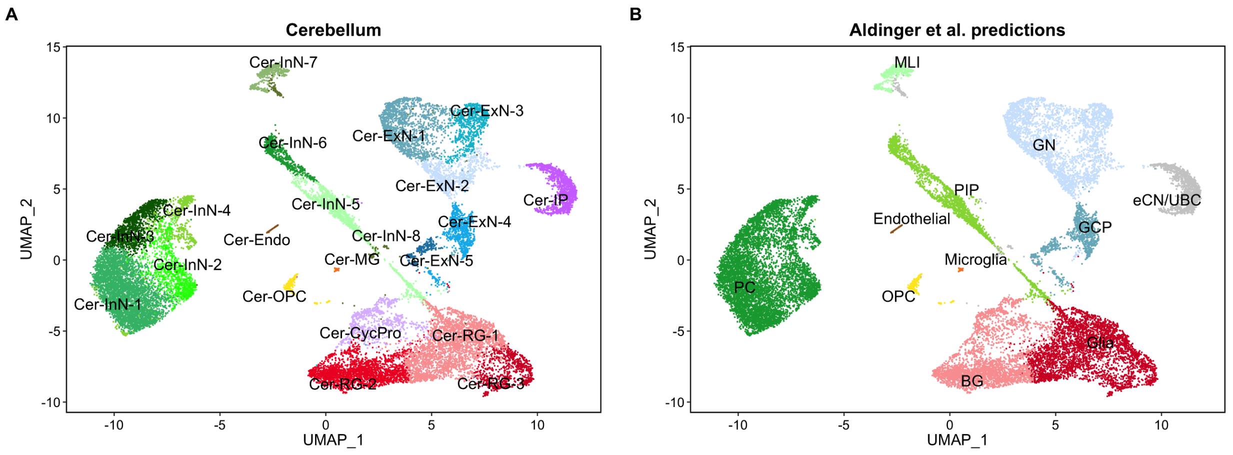

Fig. S8. Cluster annotations for fetal cerebellum. A) Labels used in this study. ExN = developing excitatory neuron; InN = developing inhibitory neuron; IP = intermediate progenitor; RG = radial glia; CycPro = cycling progenitor cells; OPC = oligodendrocyte precursor cell; Endo = endothelial cell; MG = microglia. B) Predictions based on data from the study of Aldinger et al (2021). PC = Purkinje cells; GCP = Granule cell progenitors; GN = Granule neurons; eCN/UBC = Excitatory cerebellar nuclei neurons/Unipolar brush cells; PIP = PAX2+ interneuron progenitors; BG = Bergmann glia; OPC = Oligodendrocyte precursor cells; MLI = Molecular layer interneurons.

Reference

Aldinger KA, Thomson Z, Phelps IG, Haldipur P, Deng M, Timms AE, et al. (2021): Spatial and cell type transcriptional landscape of human cerebellar development. *Nat Neurosci* 24: 1163–1175.

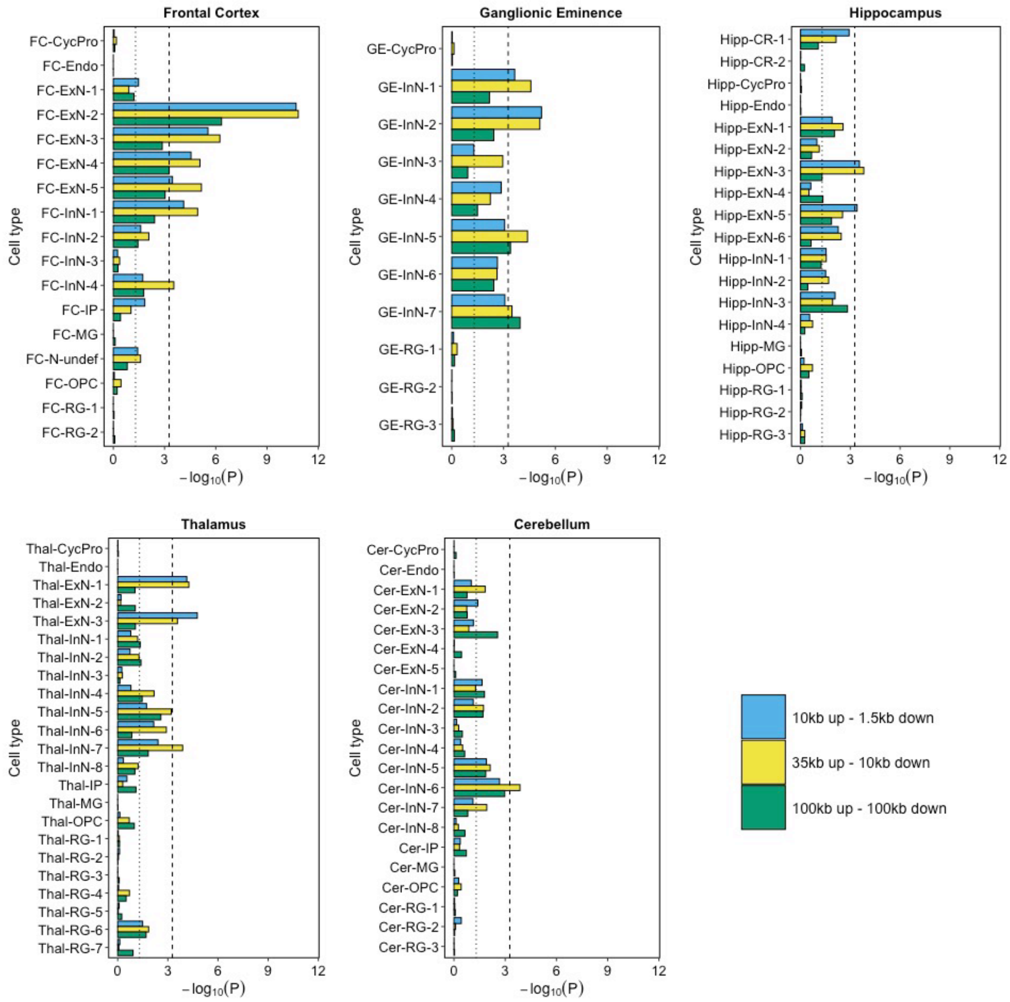

Fig. S9. MAGMA -log10 *P*-values for enrichment of genetic associations with schizophrenia (Schizophrenia Working Group of the Psychiatric Genomics Consortium, 2022) in genes in the top expression specificity decile for each identified fetal brain cell population using different extensions from the transcribed region genomic coordinates. The default extension in the MAGMA celltyping package is 10kb upstream and 1.5kb downstream of the transcribed region of each gene and this was therefore chosen for the main analyses reported in this paper. The dotted vertical line indicates nominal ($P < 0.05$) significance and the dashed vertical line indicates the Bonferroni-corrected *P*-value threshold for 91 tested cell populations ($P < 5.5 \times 10^{-4}$). ExN = developing excitatory neuron; InN = developing inhibitory neuron; CR = Cajal-Retzius cell; N-undef = neuron of undefined class; IP = intermediate progenitor; RG = radial glia; CycPro = cycling progenitor cells; OPC = oligodendrocyte precursor cell; Endo = endothelial cell; MG = microglia.

Reference

Schizophrenia Working Group of the Psychiatric Genomics Consortium (2022): Mapping genomic loci implicates genes and synaptic biology in schizophrenia. *Nature* 604: 502–508.

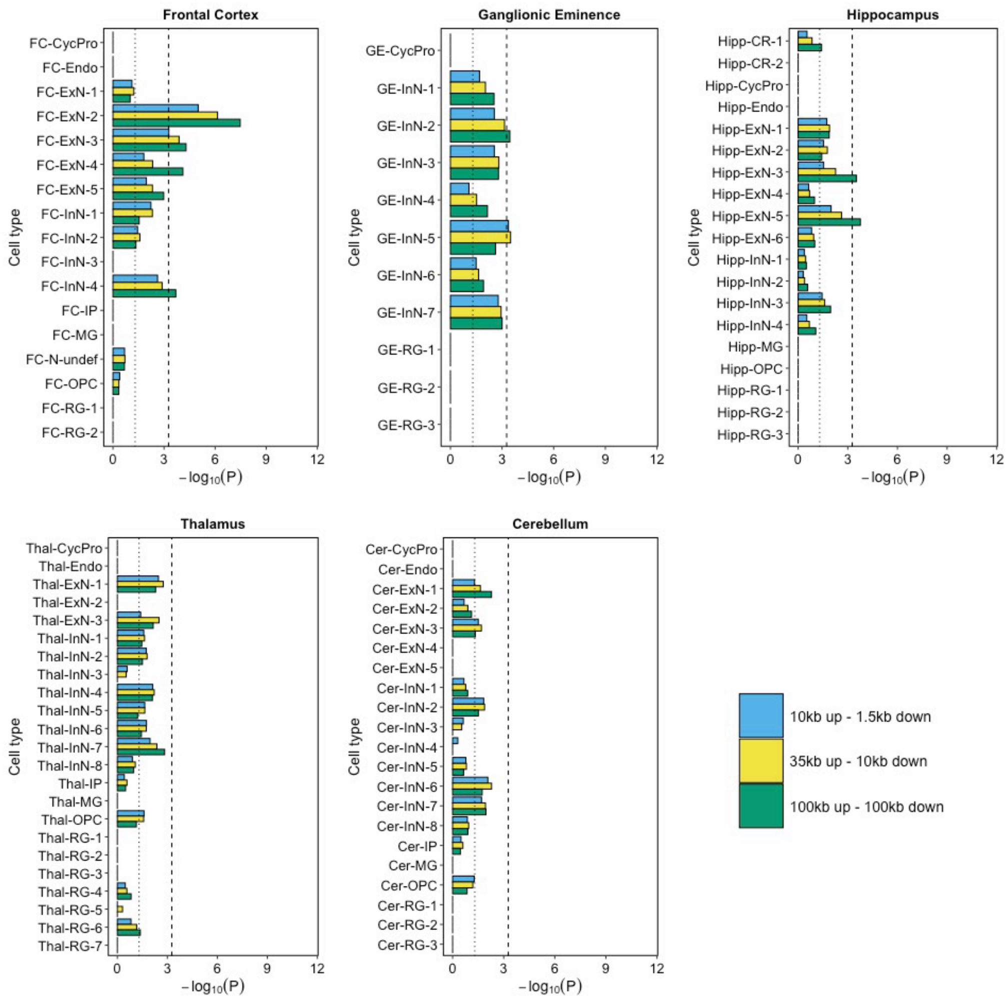

Fig. S10. sLDSR -log10 *P*-values for enrichment of genetic associations with schizophrenia (Schizophrenia Working Group of the Psychiatric Genomics Consortium, 2022) in genes in the top expression specificity decile for each identified fetal brain cell population using different extensions from the transcribed region genomic coordinates. The sLDSR authors (Finucane et al, 2018) recommend an extension of 100kb either side of the transcribed region of each gene, and this was therefore chosen for the main analyses reported in this paper. The dotted vertical line indicates nominal ($P < 0.05$) significance and the dashed vertical line indicates the Bonferroni-corrected *P*-value threshold for 91 tested cell populations ($P < 5.5 \times 10^{-4}$). ExN = developing excitatory neuron; InN = developing inhibitory neuron; CR = Cajal-Retzius cell; N-undef = neuron of undefined class; IP = intermediate progenitor; RG = radial glia; CycPro = cycling progenitor cells; OPC = oligodendrocyte precursor cell; Endo = endothelial cell; MG = microglia.

References

Schizophrenia Working Group of the Psychiatric Genomics Consortium (2022): Mapping genomic loci implicates genes and synaptic biology in schizophrenia. *Nature* 604: 502–508.

Finucane HK, Reshef YA, Anttila V, Slowikowski K, Gusev A, Byrnes A, et al. (2018): Heritability enrichment of specifically expressed genes identifies disease-relevant tissues and cell types. *Nat Genet* 50: 621–629.

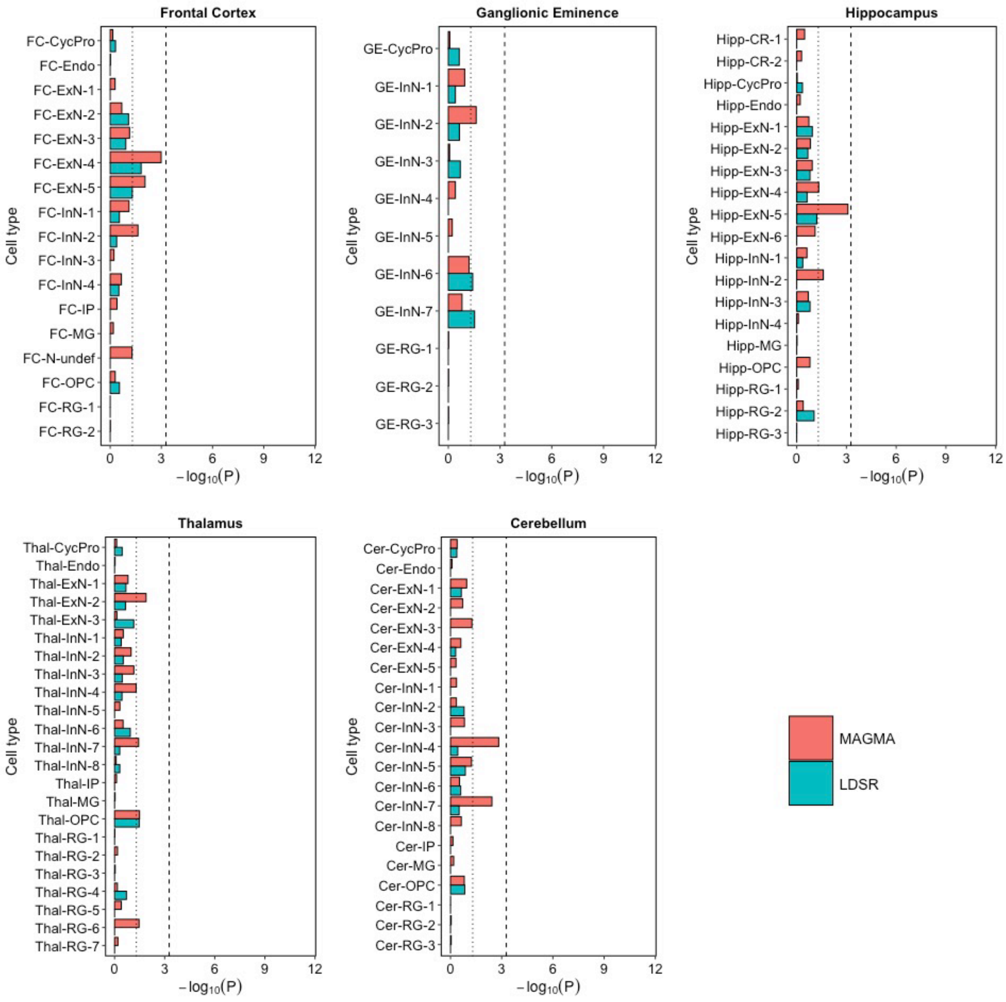

Fig. S11. -log10 *P*-values for enrichment of genetic associations with autism (Grove et al, 2019) in genes in the top expression specificity decile for each identified fetal brain cell population using MAGMA and sLDSR. The dotted vertical line indicates nominal ($P < 0.05$) significance and the dashed vertical line indicates the Bonferroni-corrected P-value threshold for 91 tested cell populations ($P < 5.5 \times 10^{-4}$). ExN = developing excitatory neuron; InN = developing inhibitory neuron; CR = Cajal-Retzius cell; N-undef = neuron of undefined class; IP = intermediate progenitor; RG = radial glia; CycPro = cycling progenitor cells; OPC = oligodendrocyte precursor cell; Endo = endothelial cell; MG = microglia.

Reference

Grove J, Ripke S, Als TD, Mattheisen M, Walters RK, Won H, et al. (2019): Identification of common genetic risk variants for autism spectrum disorder. *Nat Genet* 51: 431-444.

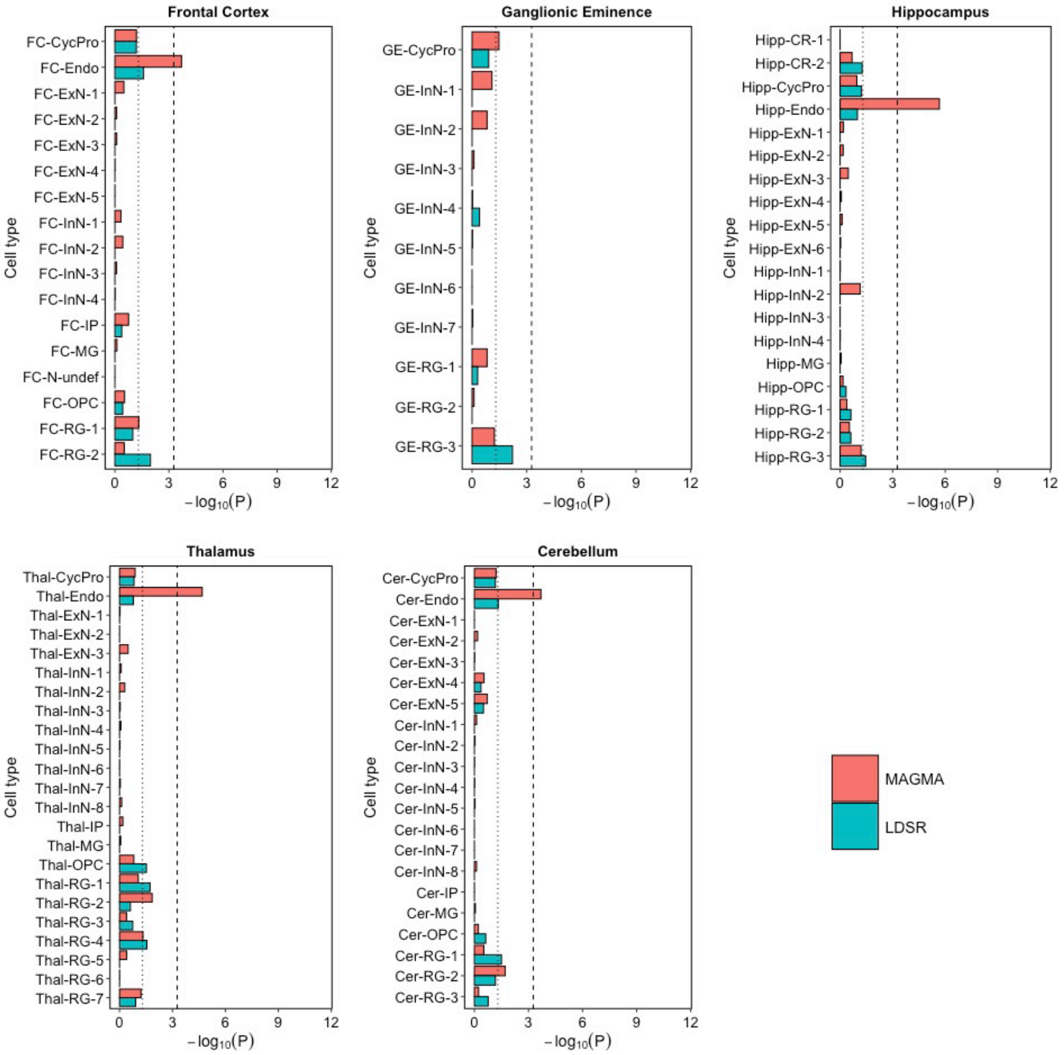

Fig. S12. -$\log_{10}$ *P*-values for enrichment of genetic associations with human height (Yengo et al, 2018) in genes in the top expression specificity decile for each identified fetal brain cell population using MAGMA and sLDSR. The dotted vertical line indicates nominal ($P < 0.05$) significance and the dashed vertical line indicates the Bonferroni-corrected *P*-value threshold for 91 tested cell populations ($P < 5.5 \times 10^{-4}$). ExN = developing excitatory neuron; InN = developing inhibitory neuron; CR = Cajal-Retzius cell; N-undef = neuron of undefined class; IP = intermediate progenitor; RG = radial glia; CycPro = cycling progenitor cells; OPC = oligodendrocyte precursor cell; Endo = endothelial cell; MG = microglia.

Reference

Yengo L, Sidorenko J, Kemper KE, Zheng Z, Wood AR, Weedon MN, et al. (2018): Meta-analysis of genome-wide association studies for height and body mass index in ~700000 individuals of European ancestry. *Hum Mol Genet* 27: 3641–3649.

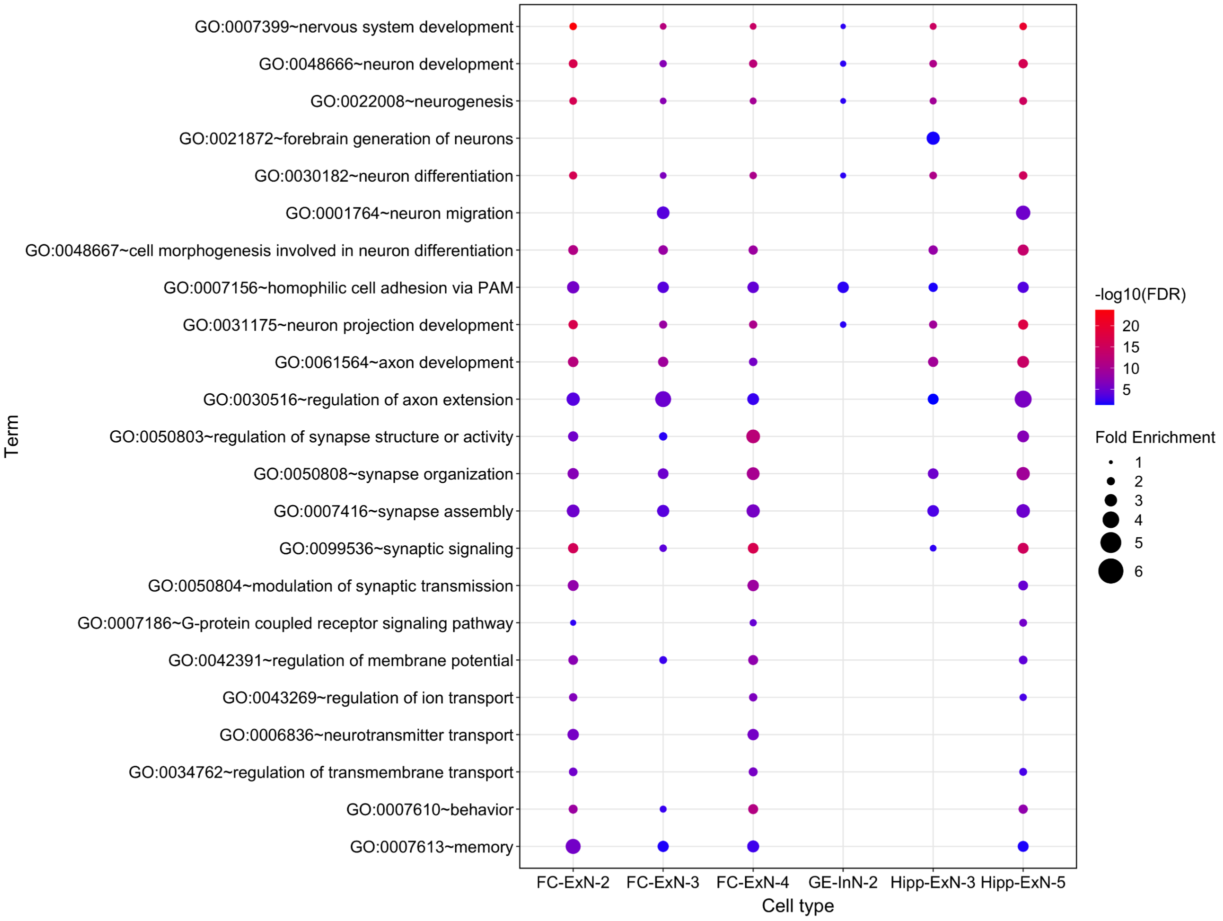

Fig. S13. Gene Ontology (GO) biological process enrichment analysis of genes in the top expression specificity decile of 6 fetal neuron populations implicated in common variant genetic liability to schizophrenia by MAGMA / sLDSR at the Bonferroni significance threshold. Enrichments were tested against terms included in the comprehensive GO_BP_FAT category in the DAVID Bioinformatics Resource 6.8, using background lists of all genes detected in each cell population. We show the top 20 terms for each implicated cell population, where enrichment FDR < 0.05. For highly similar GO terms showing the same level of enrichment within and across cell populations, we list only one representative term. ExN = developing excitatory (glutamatergic) neuron; InN = developing inhibitory (GABAergic) neuron; FC = frontal cortex; GE = ganglionic eminence; Hipp = hippocampus.

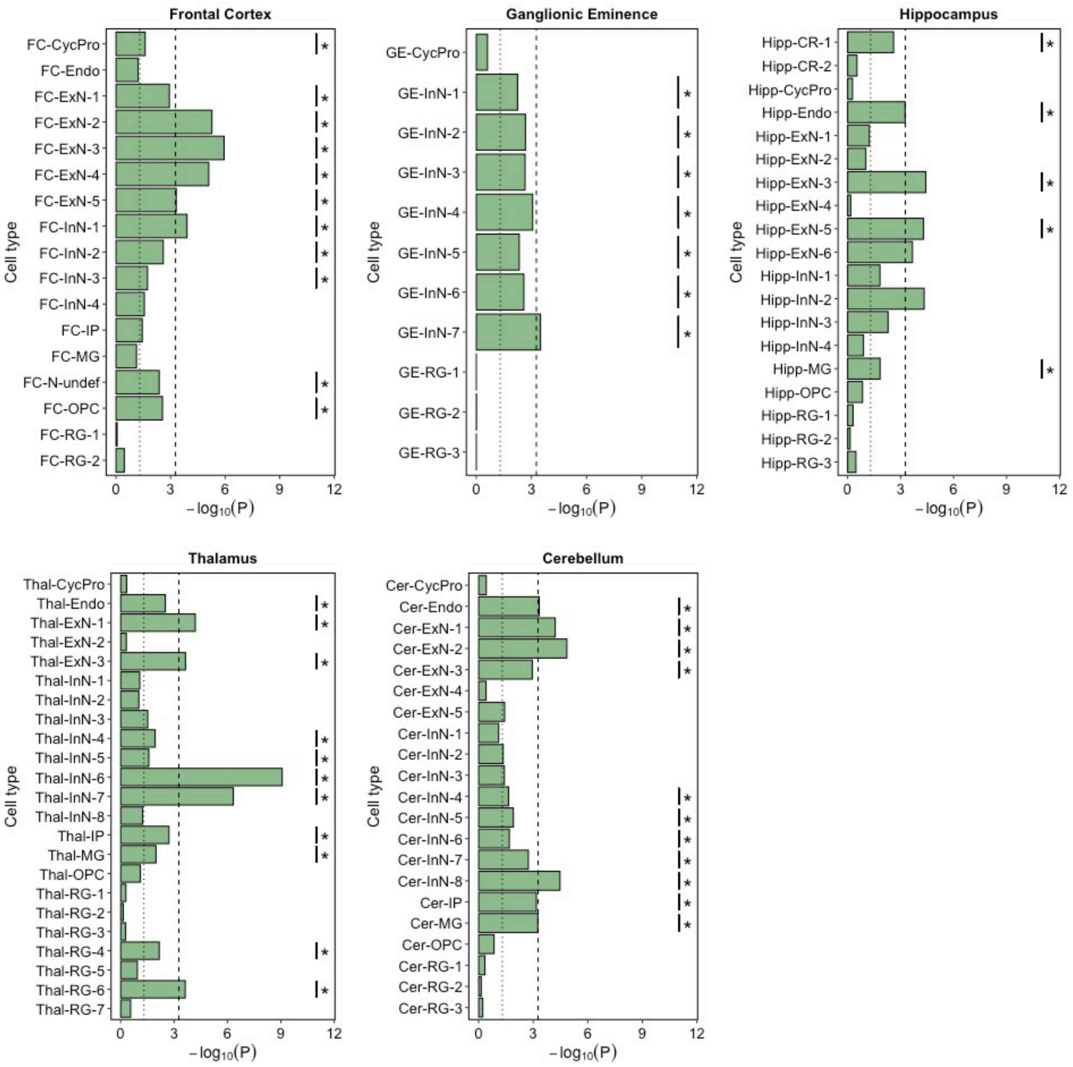

Fig. S14. Wilcoxon rank sum test -$\log_{10}$ *P*-values for higher gene expression specificity of 78 genes carrying an excess (FDR < 0.05) of rare damaging coding variation in autism (Satterstrom et al, 2020) in cell populations of the human fetal brain. The dotted vertical line indicates nominal (*P* < 0.05) significance, the dashed vertical line indicates the Bonferroni-corrected *P*-value threshold for 91 tested cell populations ($P < 5.5 \times 10^{-4}$) and asterisks indicate cell populations in which the 32 genes have higher expression specificity at FDR < 0.05. ExN = developing excitatory neuron; InN = developing inhibitory neuron; CR = Cajal-Retzius cell; N-undef = neuron of undefined class; IP = intermediate progenitor; RG = radial glia; CycPro = cycling progenitor cells; OPC = oligodendrocyte precursor cell; Endo = endothelial cell; MG = microglia.

Reference

Satterstrom FK, Kosmicki JA, Wang J, Breen MS, De Rubeis S, An JY, et al. (2020): Large-Scale Exome Sequencing Study Implicates Both Developmental and Functional Changes in the Neurobiology of Autism. *Cell* 180: 568-584.

Supplementary Methods

Nuclei preparation and single nuclei RNA Sequencing (snRNA-seq)

Optimization experiments on cryopreserved cell suspensions indicated superior sequencing data (in terms of reads in 'cells') when nuclei rather than intact cells were used as input for library preparation, and we therefore chose single nuclei RNA Sequencing (snRNA-seq) as the basis of this study. We note that snRNA-seq has been used in nearly all existing studies of adult human brain (e.g. 1-3), which have largely utilized frozen tissue, and has been shown to yield a similar resolution of brain cell populations to scRNA-seq (4) while potentially limiting biases arising from differences in cell size, morphology and viability (5). However, Skene and colleagues (6) found that RNA transcripts destined for dendrites were under-represented in snRNA-Seq (compared with scRNA-Seq) data from the adult human brain, and therefore use of snRNA-Seq might have limited detection of these transcripts within more mature neurons in the present study.

Cryopreserved cell suspensions from dissected brain regions from each fetus were defrosted and gently lysed to release nuclei. Large debris and unlysed cells were pelleted by centrifugation and the supernatant strained through a 10µm filter (Cambridge Bioscience). Nuclei were examined under a light microscope to ensure integrity and nuclei numbers determined using an Invitrogen Countess II (ThermoFisher Scientific). Nuclei were pelleted by centrifugation and then resuspended in buffer (1X PBS, 1% BSA, 0.2U/µl RNAse inhibitor) to a concentration of 1000 nuclei/µl. snRNA-seq libraries were prepared from ~10,000 nuclei from each sample using Chromium Single Cell 3′ (v3) reagents (10X Genomics). Quality control of libraries was performed using the Agilent 5200 Fragment Analyzer before sequencing on an Illumina NovaSeq 6000 to a depth of at least 865 million (median = 1.01 billion) read pairs per library.

<u>snRNA-seq data processing</u>

Raw sequencing data were converted into FASTQ files using *bcl2fastq* (Illumina, version 2.18). snRNA-seq reads were aligned to the hg38 build of the human reference genome (10X Genomics, refdata-gex-GRCh38-2020-A) and quantified using *cellranger count* (10X Genomics, version 5.0.1) with the *include-introns* flag added to incorporate reads derived from unspliced RNA fragments in the quantification process. This produced a filtered matrix of gene expression counts, a gene annotation file and a file of cell barcodes for each sample which were read into R 4.0.3 using *DropletUtils::add10Xcounts* (7,8). A stringent quality control procedure was carried out to ensure that only high-quality single-nuclei data were processed. Doublets were identified using *scDblFinder::scDblFinder* (9,10) and per cell and per gene quality control metrics were generated using the functions *scater::addPerCellQC* and *scater::addPerFeatureQC* respectively (11). Cells were excluded if they expressed fewer than 1000, or greater than 5000, genes, if greater than 5% or 10% of their transcriptome mapped to the mitochondrial genome or ribosomal genes respectively, or if they were identified as a doublet. Genes were excluded from the analysis if they were expressed in fewer than 3 cells. *MALAT1* and genes from the mitochondrial genome were also removed. Nuclei retained for downstream analyses had a median of 4202 unique molecular identifier (UMI) counts per cell.

Subsequent snRNA-seq data processing steps from normalization through to cluster visualization were carried out using Seurat, version 4.0.3 (12). To account for variability in sequencing depth and technical artefacts that can be introduced into snRNA-seq data during sample preparation (13), the data were normalized by dividing the UMI count of each gene in each cell by the total number of UMIs in that cell, multiplying these ratios by a scale factor of 10,000 and performing a log-transformation (*Seurat::NormalizeData*). Next, the top 2000 most variable genes in the dataset were identified (*Seurat::FindVariablesFeatures*) and all normalized gene expression values were scaled such that mean

expression and variance across all cells equalled 1 and 0 respectively (*Seurat::ScaleData*). To reduce the dimensionality of the dataset a principal component analysis was run on the scaled gene expression values of the top 2000 most variable genes (*Seurat::RunPCA*). Cells were then clustered using a 2-step approach. First, a shared-nearest neighbour (SNN) graph was created using the first 17 principal components in the data (*Seurat::FindNeighbors*). Here, the k-nearest neighbours of each cell (k=30) were identified in the PCA space and these neighbours were ranked based on how close they were to the original cell. Then, an SNN graph was built by adding edges between pairs of cells if they have one or more nearest neighbours in common. Edges were weighted based on the ranking of the common neighbour cell(s). Second, the *Seurat::FindClusters* function was run which iteratively grouped cells into distinct communities until the algorithm had maximized the number of weighted edges that exist within communities as opposed to between communities. This returned a set of clusters for each brain region, where cells in individual clusters had similar gene expression profiles. Clusters were visualized in 2D space using Uniform Manifold Approximation and Projection (UMAP) (14) using the function *Seurat::Dimplot*. At this stage, an iterative procedure was employed to remove clusters where cell populations derived disproportionately from a single donor. If 2 or more donors did not contribute at least 5 cells to a single cluster, then the cells in that cluster were removed from the analysis and the steps from dimensionality reduction through to cluster visualization were re-run until all clusters across all brain regions passed this exclusion criterion.

<u>snRNA-seq cluster stability</u>

To assess cluster stability, a 5-fold cross validation procedure was carried out using the scRNA-seq benchmarking tool (15). First, the raw UMI counts for each brain region were normalized and sub-divided into 5 folds (separate instances of a test and training dataset). Each fold was stratified to maintain equal cell proportions of each cluster. Then, an intra-dataset cross validation was run using a

supervised support vector machine (SVM) classifier with a linear kernel that predicted the cluster identities in the test data based on the identities specified in the training data. The true and predicted identities of clusters in the test data were compared and an F1 accuracy score was calculated for each cluster. The F1 scores for all clusters in each brain region are shown in Supplementary table S1.

Assigning cell-type identity to snRNA-seq clusters

We primarily assigned cell-type identity to each cluster based on expression of known cell marker genes (Supplementary Figures 1-5) and other differentially-expressed genes (upregulated in each cluster compared to all other clusters from that brain region) which were identified using *Seurat::FindAllMarkers* (Supplementary tables S2-S6). Where public scRNA-seq / snRNA-seq datasets where available from the human fetal brain region assayed, a cell label transfer approach was also implemented in which a reference dataset was created from the pre-existing data and the cell type labels in the reference data were projected onto cells in the query dataset based on the similarity of their gene expression profiles. For the frontal cortex, we used existing scRNA-seq data from the fetal cerebral cortex generated by Nowakowski et al (16) and Polioudakis et al (17) to corroborate our cell annotations. The raw gene expression count matrix and cell metadata from the study of Polioudakis and colleagues (17) were downloaded from http://solo.bmap.ucla.edu/shiny/webapp/. These data were then normalized, the top 2000 features identified and scaled as described above. To identify transfer anchors between the datasets, *Seurat::FindTransferAnchors* was run. This implemented a principal component analysis on the reference dataset onto which the clusters for each brain region in this study were separately projected. These data were L2-normalized and then for each cell in the query dataset the k-nearest neighbours for that cell in the reference dataset were identified, and vice versa. Finally, high confidence transfer anchors - pairs of cells (one from each dataset) with mutual nearest neighbours in opposing datasets - were established and scored based on the shared neighbour overlap between the

anchor cells and query cells (18,19). These anchors were then used to transfer the cell labels from cells in the reference dataset onto similar cells in the query dataset by running *Seurat::TransferData* and visualised via UMAP (Supplementary Figure S6). For the Nowakowski et al. study (16), clustifyr (20) was used for label transfer. A pre-compiled clustifyr-compatible reference gene expression matrix for the Nowakowski et al study was downloaded (from https://github.com/rnabioco/clustifyrdata/raw/master/data/ref_cortex_dev.rda) and a Spearman ranked correlation was performed to quantify the similarity of the normalized transcriptome profiles in clusters in the query data to that in the reference data. Query clusters for fetal frontal cortex and ganglionic eminence were assigned the identity of the highest correlated reference cell type and clusters were visualized by UMAP (Supplementary Figures S6 and S7). The clustifyr package provides additional functionality for comparison of signature marker genes in cells/clusters across datasets that can be used for cell type classification. For the fetal cerebellum, we were therefore able to use published lists of differentially expressed genes for each cell type generated by Aldinger and colleagues (21) for corroboration of cell annotations (Supplementary Figure S8).

References (Supplementary Methods)

1. Lake BB, Ai R, Kaeser GE, Salathia NS, Yung YC, Liu R, et al. (2016): Neuronal subtypes and diversity revealed by single-nucleus RNA sequencing of the human brain. *Science* 352: 1586-1590.
2. Agarwal D, Sandor C, Volpato V, Caffrey TM, Monzón-Sandoval J, Bowden R, et al. (2020): A single-cell atlas of the human substantia nigra reveals cell-specific pathways associated with neurological disorders. *Nat Commun* 11: 4183

3. Bakken TE, Jorstad NL, Hu Q, Lake BB, Tian W, Kalmbach BE, et al. (2021): Comparative cellular analysis of motor cortex in human, marmoset and mouse. *Nature* 598: 111-119.
4. Bakken TE, Hodge RD, Miller JA, Yao Z, Nguyen TN, Aevermann B, et al. (2018): Single-nucleus and single-cell transcriptomes compared in matched cortical cell types. *PLoS One* 13: e0209648.
5. Armand EJ, Li J, Xie F, Luo C, Mukamel EA (2021): Single-Cell Sequencing of Brain Cell Transcriptomes and Epigenomes. *Neuron* 109: 11-26.
6. Skene NG, Bryois J, Bakken TE, Breen G, Crowley JJ, Gaspar HA, et al. (2018): Genetic identification of brain cell types underlying schizophrenia. *Nat Genet* 50: 825–833.
7. Lun ATL, Riesenfeld S, Andrews T, Dao TP, Gomes T, Marioni JC, et al. (2019): EmptyDrops: distinguishing cells from empty droplets in droplet-based single-cell RNA sequencing data. *Genome Biol* 20: 63.
8. Griffiths JA, Richard AC, Bach K, Lun ATL, Marioni JC (2018): Detection and removal of barcode swapping in single-cell RNA-seq data. *Nat Commun* 9: 2667.
9. Germain PL, Lun A, Macnair W, Robinson MD (2021): Doublet identification in single-cell sequencing data using scDblFinder. *F1000Research* 10: 979.
10. Xi NM, Li JJ (2021): Benchmarking Computational Doublet-Detection Methods for Single-Cell RNA Sequencing Data. *Cell Syst* 12: 176-194.
11. McCarthy DJ, Campbell KR, Lun ATL, Wills QF (2017): Scater: pre-processing, quality control, normalization and visualization of single-cell RNA-seq data in R. *Bioinformatics* 33: 1179–1186.

12. Hao Y, Hao S, Andersen-Nissen E, Mauck WM, Zheng S, Butler A, et al. (2021): Integrated analysis of multimodal single-cell data. *Cell* 184: 3573-3587.

13. Stegle O, Teichmann SA, Marioni JC (2015): Computational and analytical challenges in single-cell transcriptomics. *Nat Rev Genet* 16: 133–145.

14. Becht E, McInnes L, Healy J, Dutertre C-A, Kwok IWH, Ng LG, et al. (2019): Dimensionality reduction for visualizing single-cell data using UMAP. *Nat Biotechnol* 37: 38–44.

15. Abdelaal T, Michielsen L, Cats D, Hoogduin D, Mei H, Reinders MJT, et al. (2019): A comparison of automatic cell identification methods for single-cell RNA sequencing data. *Genome Biol* 20: 194.

16. Nowakowski TJ, Bhaduri A, Pollen AA, Alvarado B, Mostajo-Radji MA, Di Lullo E, et al. (2017): Spatiotemporal gene expression trajectories reveal developmental hierarchies of the human cortex. *Science* 358: 1318–1323.

17. Polioudakis D, de la Torre-Ubieta L, Langerman J, Elkins AG, Shi X, Stein JL, et al. (2019): A Single-Cell Transcriptomic Atlas of Human Neocortical Development during Mid-gestation. *Neuron* 103: 785–801.

18. Stuart T, Butler A, Hoffman P, Hafemeister C, Papalexi E, Mauck III WM, et al. (2019): Comprehensive Integration of Single-Cell Data. *Cell* 177: 1888-1902.

19. Haghverdi L, Lun ATL, Morgan MD, Marioni JC. (2018): Batch effects in single-cell RNA-sequencing data are corrected by matching mutual nearest neighbors. *Nat Biotechnol* 36: 421–427.

20. Fu R, Gillen AE, Sheridan RM, Tian C, Daya M, Hao Y, et al. (2020): clustifyr: an R package for automated single-cell RNA sequencing cluster classification. *F1000Research* 9: 223.

21. Aldinger KA, Thomson Z, Phelps IG, Haldipur P, Deng M, Timms AE, et al. (2021): Spatial and cell type transcriptional landscape of human cerebellar development. *Nat Neurosci* 24: 1163–1175.
